# The neuro-pathophysiology of temporomandibular disorders-related pain: a systematic review of structural and functional MRI studies

**DOI:** 10.1186/s10194-020-01131-4

**Published:** 2020-06-19

**Authors:** Yuanyuan Yin, Shushu He, Jingchen Xu, Wanfang You, Qian Li, Jingyi Long, Lekai Luo, Graham J. Kemp, John A. Sweeney, Fei Li, Song Chen, Qiyong Gong

**Affiliations:** 1 Huaxi MR Research Center (HMRRC), Department of Radiology, West China Hospital of Sichuan University, Chengdu, 610041 Sichuan People’s Republic of China; 2 State Key Laboratory of Oral Diseases, National Clinical Research Center for Oral Diseases, Department of Orthodontics, West China School of Stomatology, Sichuan University, Chengdu, 610041 Sichuan People’s Republic of China; 3 Psychoradiology Research Unit of Chinese Academy of Medical Sciences, Functional and Molecular Imaging Key Laboratory of Sichuan Province, West China Hospital of Sichuan University, Chengdu, 610041 Sichuan People’s Republic of China; 4 Liverpool Magnetic Resonance Imaging Centre (LiMRIC) and Institute of Ageing and Chronic Disease, University of Liverpool, Liverpool, UK; 5 Department of Psychiatry, University of Cincinnati, Cincinnati, OH USA

**Keywords:** Temporomandibular disorders, Chronic pain, Magnetic resonance imaging, Gray matter, White matter, Brain structure and function, Splint therapy Psychoradiology

## Abstract

Chronic pain surrounding the temporomandibular joints and masticatory muscles is often the primary chief complaint of patients with temporomandibular disorders (TMD) seeking treatment. Yet, the neuro-pathophysiological basis underlying it remains to be clarified. Neuroimaging techniques have provided a deeper understanding of what happens to brain structure and function in TMD patients with chronic pain. Therefore, we performed a systematic review of magnetic resonance imaging (MRI) studies investigating structural and functional brain alterations in TMD patients to further unravel the neurobiological underpinnings of TMD-related pain. Online databases (PubMed, EMBASE, and Web of Science) were searched up to August 3, 2019, as complemented by a hand search in reference lists. A total of 622 papers were initially identified after duplicates removed and 25 studies met inclusion criteria for this review. Notably, the variations of MRI techniques used and study design among included studies preclude a meta-analysis and we discussed the findings qualitatively according to the specific neural system or network the brain regions were involved in. Brain changes were found in pathways responsible for abnormal pain perception, including the classic trigemino-thalamo-cortical system and the lateral and medial pain systems. Dysfunction and maladaptive changes were also identified in the default mode network, the top-down antinociceptive periaqueductal gray-raphe magnus pathway, as well as the motor system. TMD patients displayed altered brain activations in response to both innocuous and painful stimuli compared with healthy controls. Additionally, evidence indicates that splint therapy can alleviate TMD-related symptoms by inducing functional brain changes. In summary, MRI research provides important novel insights into the altered neural manifestations underlying chronic pain in TMD.

## Introduction

Temporomandibular disorders (TMD) are a subgroup of craniofacial pain disorders involving pain and dysfunction of the temporomandibular joint (TMJ), masticatory muscles and associated musculoskeletal structures of the head and neck. It is the commonest cause of nondental pain in the orofacial region [1]. Patients with TMD frequently suffer from localized pain, impaired jaw movement and noise from TMJ during jaw movement, as well as less specific symptoms including ear pain and stuffiness, tinnitus, dizziness, neck pain and headache [2, 3]. Although not life-threatening, it can affect oral health-related quality of life, and the symptoms can be chronic and difficult to manage [4, 5]. However, as with many chronic pain syndromes, the neurobiological mechanisms pertaining to pain in TMD remain to be clarified.

Initially, TMD-related chronic pain was considered to be caused primarily by peripheral factors, such as chronic inflammation of the TMJ, microtrauma of the masticatory muscles, and oromotor dysfunction. However, the correlation between pain severity and tissue pathology is often poor, and not all patients have clearly identifiable peripheral etiological factors [6–8]. Moreover, TMD patients may have pain in other body areas [9, 10], and there is high comorbidity with functional syndromes such as fibromyalgia [11] and irritable bowel syndrome [12]. Such evidence implicates central nervous system dysfunction in pain associated with TMD [13–15].

Previous studies have thoroughly investigated changes in brain activity when experiencing clinical pain [16]. Persistent nociceptive input to brain can induce maladaptive anatomical and physiological changes in the brain via pathology or compensation [17, 18]. Functional and structural magnetic resonance imaging (MRI) methods have been widely used separately or combined to explore brain alterations in patients with chronic pain [19–21], including TMD, for better understanding of the neural mechanisms of pain perception and chronification. MRI studies in TMD have provided evidence for structural and functional changes within the ascending trigemino-thalamo-cortical pathway involving the trigeminal nerve root, spinal tract subnucleus caudalis (SpVc), thalamus, and primary somatosensory cortex (S1). For example, SpVc, which lies within the caudal brainstem and processes orofacial nociceptive input from the trigeminal peripheral nerve ending, shows decreased gray matter volume (GMV) [22] and elevated cerebral blood flow (CBF) [23] in TMD patients. Brain alterations have also been found in widespread brain regions involved in the lateral and medial pain systems, the default mode network (DMN) and the top-down antinociceptive periaqueductal gray (PAG)-raphe magnus pathway functioning for pain perception and modulation. These new findings of structural and functional alterations at network levels may either account for the pathogenesis or be the consequences of pain in TMD. Although there are systematic reviews and meta-analysis on brain alterations of patients with orofacial pain disorders within which TMD was involved [24–26], there has so far been no comprehensive review specifically focusing on TMD to highlight progress in this area of work.

Therefore, this systematic review was conducted to provide an overview of neuroimaging MRI studies that shed light on neuro-pathophysiological basis underlying TMD-related pain by defining associated structural and functional brain alterations. Studies reporting brain changes after splint treatment that have potential implications for clarifying the therapeutic mechanism of TMD were also reviewed. Finally, while reviewing progress in this field, consideration was also given to describing the limitations of previous work and suggesting future directions for neuroimaging investigations of TMD.

## Methods

### Search strategy and study selection

We followed the Preferred Reporting Items for Systematic reviews and Meta-Analyses (PRISMA) guidelines for data collection [27]. Studies were identified by searching electronic databases including PubMed, EMBASE, and Web of Science up to August 3, 2019. The following search terms were used: (TMD OR temporomandibular disorders) AND pain AND (neuroimaging OR (sMRI OR structural MRI) OR (DTI OR diffusion tensor imaging) OR (fMRI OR functional MRI) OR (ASL OR arterial spin labeling) OR (MRS OR magnetic resonance spectroscopy)). Additional publications were identified by manual search in reference lists. Studies were included according to the following criteria: 1) original publications in English from peer-reviewed journals; 2) studies conducted in patients with TMD diagnosed with Research Diagnostic Criteria for TMD (RDC/TMD) [28] or Diagnostic Criteria for TMD (DC/TMD) [29]; and 3) MRI studies exploring the brain structure and function of TMD patients. Exclusion criteria included: 1) conference abstracts, theoretical papers, and reviews; and 2) studies using magnetoencephalography or electroencephalography. Two of us (Y.Y.Y. and S.S.H.) independently conducted the literature search. The results of these two searches were compared and any inconsistencies were discussed, and a consensus decision reached about inclusion.

### Quality assessment

A customized 9-point checklist based on the Strengthening the Reporting of Observational Studies in Epidemiology statement [30] was used for study quality and risk of bias assessment (Additional file 1). The checklist focused on characteristics of participants for considerations of the heterogeneity of diagnosis and the impact of medication and comorbidity on results. We also incorporated items assessing the imaging methodology and statistical analysis. Any study scoring ≥5 was included in this systematic review. Two independent reviewers (Y.Y.Y. and S.S.H.) performed the assessment and disagreements were resolved by discussion to reach a consensus.

### Data analysis

The present results of brain structural and functional differences between patients with TMD and controls were qualitatively described according to the specific brain system or network the brain regions were involved in. Altered brain activations in response to mechanical stimuli, the effects on the brain of splint treatment, as well as the association between brain alterations and other pain-related measurements were summarized and presented descriptively.

## Results

### Search results and study characteristics

**Fig. 1:**
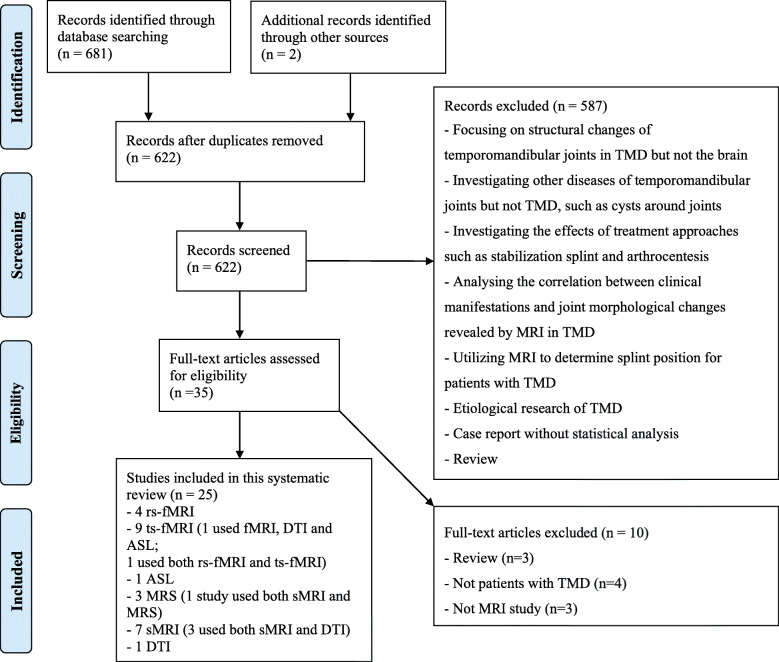
Flowchart of the selection procedure. *Abbreviations:* TMD, temporomandibular disorders; MRI, magnetic resonance imaging; fMRI, functional MRI; sMRI, structural MRI; rs-fMRI, resting-state fMRI; ts-fMRI, task-state fMRI; ASL, arterial spin labeling; MRS, magnetic resonance spectroscopy; DTI, diffusion tensor imaging

Figure 1 shows a flowchart of the search and selection process. A total of 25 studies published between January 1, 2010 and August 3, 2019 were included (details in Tables 1 and 2 and Additional file 2). Twenty-four MRI studies [22, 23, 31, 32, 35, 39, 41–43, 45–59] compared patients with TMD and healthy controls, while 1 longitudinal study recruited only patients [60].

**Table 1:** Summary of functional MRI studies on TMD, MRI techniques including rs-fMRI, ts-fMRI, ASL, and MRS

Studies	Modality	Analysis method	Patients					Controls	Main findings in TMD patients compared to HC
			Characteristics	Number and age^a^	Duration^a^	Medication (n)	Clinical assessments	Number and age^a^	
**rs-fMRI (*n*** **= 4)**									
Kucyi et al. [46]	rs-fMRI	Voxel-wise FC	TMD (RDC/TMD)	17F; 33.1 ± 11.9 y	9.3 ± 8.3 (0.75–30) y	8	NPS^1^, Pain Catastrophizing Scale [44]	17F; 32.2 ± 10.2 y	In TMD: • Increased mPFC FC with other DMN regions, including PCC/PCu, retrosplenial cortex and areas within visual cortex • Pain rumination scores positively correlated to mPFC FC with the PCC/PCu, retrosplenial cortex, medial thalamus and PAG
He et al. [47]	rs-fMRI	Whole brain fALFF	TMD (RDC/TMD)	9M, 14F; 22.4 ± 3.6 y; 11/23 received 3-month splint therapy	14.8 ± 20.7 months	0	SCL-90, GCPS [83], Helkimo indices [78], CR–MI discrepancy index [76]	9M, 11F; 23.1 ± 2.4 y	In TMD: • Decreased fALFF in left precentral gyrus, SMA, middle frontal gyrus, and right OFC • Negative correlation between fALFF in left precentral gyrus and vertical CR-MI discrepancy • Improved symptoms and signs after treatment, with increased fALFF in left precentral gyrus and left posterior insula compared with pretreatment
He et al. [48]	rs-fMRI	Voxel-wise FC	TMD (RDC/TMD)	11M, 19F; 22.1 ± 3.8 (15–29) y; 16/30 had myofascial pain	17.3 ± 22.4 months	0	SCL-90, GCPS [83], Helkimo indices [78]	9M, 11F; 23.1 ± 2.4 (20–30) y	In TMD: • Decreased FC in ventral corticostriatal circuitry, between ventral striatum and ventral frontal cortices, including ACC and anterior IC • Decreased FC in dorsal corticostriatal circuitry, between dorsal striatum and dorsal cortices, including precentral gyrus and supramarginal gyrus • Decreased FC within striatum • Decreased corticostriatal FC correlated with clinical measurements including Di and pain intensity
Zhang et al. [49]	rs-fMRI	ReHo and voxel-wise FC	TMD synovitis (RDC/TMD)	8F; 33.5 ± 8.7 y	NA	0	VAS^2^	10F; 33.9 ± 7.3 y	In TMD: • Decreased regional homogeneity in right anterior IC • Decreased positive FC between right anterior IC and MCC • Decreased negative FC between right anterior IC and the precuneus
**ts-fMRI (*n* = 9)**									
Nebel et al. [52]	ts-fMRI	Brain activation	TMD (RDC/TMD)	13F; 28.7 ± 7.6 y	NA	NA	SF-MPQ [33]	12F; 28.8 ± 7.9 y	In TMD: • Distinct subregions of contralateral S1, S2 and IC responded maximally for TMD and HC • Primary auditory cortex activation • Greater activations bilaterally in ACC and contralaterally in the amygdala
Ichesco et al. [51]	rs-fMRI and ts-fMRI	Voxel-wise FC	Myofascial TMD (RDC/TMD)	8F; 23–31 y	NA	NA^b^	VAS^2^, SF-MPQ [33], STPI [34]	8F; 22–27 y	In TMD: • Increased FC between left anterior IC and pgACC during both resting state and applied pressure pain • Negative correlation between anterior IC-ACC connectivity and clinical pain intensity by VAS
Wessman et al. [54]	ts-fMRI	Brain activation and ROI-wise FC	TMD (RDC/TMD)	17F; 35.2 ± 11.6 y	9.3 ± 8.3 (0.75–30) y	8	NRS^3^	17F; 34 ± 9.9 y	In TMD: • Slow reaction times for all Stroop tasks • Increased task-evoked responses in brain areas implicated in attention (lateral prefrontal, inferior parietal), emotional processes (amygdala, pgACC), motor planning and performance (SMA and M1), and activations of the DMN (mPFC and PCC) • Decreased FC between prefrontal and cingulate cortices and between amygdala and cingulate cortex
Zhao et al. [53]	ts-fMRI	Brain activation	TMD synovitis with unilateral biting pain (RDC/TMD)	3M, 11F; 33.7 ± 13.2 y; contralateral (*n* = 8) and ipsilateral (*n* = 6) TMD biting pain	NA	NA	VAS^2^, SCL-90	7M, 7F; 23.7 ± 0.9 y	TMJ synovitis patients with contralateral or ipsilateral biting pain showed activations in inferior frontal gyrus, superior temporal gyrus, medium frontal gyrus, M1, and ACC; of these ACC was not activated in HC
Gustin et al. [50]	ts-fMRI; DTI; ASL	Brain activation, FA, and CBF	TMD (RDC/TMD)	4M, 13F; 44 ± 3 y	10.7 ± 2.9 y	13	VAS^2^, MPQ [36]	26M, 27F; 41 ± 2 y	Positive results for PTN patients, but not TMD: • Showed S1 functional reorganization • Showed reduced CBF in contralateral S1 • Showed decreased FA in contralateral S1
Lickteig et al. [60]	ts-fMRI	Brain activation	TMD (RDC/TMD)	1M, 13F; 25.7 ± 8.7 (21–53) y	NA	NA	GCPS [83], Mandibular Function Impairment Questionnaire [131]	No control group	In TMD: • Subjective pain ratings decreased, and symmetry of condylar movements increased during therapy • fMRI during occlusion showed activation decrease in right anterior IC and right cerebellum during therapy • Correlation analysis between pain score and fMRI activation decrease identified right anterior IC, left posterior IC, and left cerebellar hemisphere • Left cerebellar and right M1 activation magnitude negatively associated with symmetry of the condylar movements
He et al. [57]	ts-fMRI	Brain activation	TMD (RDC/TMD)	11M, 19F; 22.1 ± 3.8 (15–29) y; 16/30 with myofascial pain	17.3 ± 22.4 months	0	SCL-90, GCPS [83], Helkimo indices [78]	9M, 11F; 23.1 ± 2.4 (20–30) y	• TMD showed decreased positive activity in left M1, right and left inferior temporal gyrus, and left cerebellum, and increased negative activations in the right mPFC during teeth clench • For the 11 TMD after splint treatment, these areas returned to normal neural activity
Harper et al. [56]	ts-fMRI	Brain activation	Myofascial TMD (RDC/TMD)	1M, 9F; 24.9 ± 1.2 y	2.3 ± 2.0 y	NA^b^	VAS^2^, SF-MPQ [33]	10F; 26.9 ± 4.4 y	• SVM could determine location of pain evoked from pressure on temporalis and thumb in TMD, but not in HC • Differences in TMD included decreased responses to temporalis-evoked pain in the left OFC, ACC, and operculum • No significant difference in pain-evoked BOLD response for a location remote from the TMJ (the thumb)
Roy et al. [55]	ts-fMRI	Brain activation	TMD with jaw pain (TMD pain screening questionnaire [132])	6M, 10F; 36.56 (18–68) y	≥ 6 months	NA	GCPS [83], VAS^2^	6M, 9F; 30.5 (18–58) y	• For controlled grip-force task, SVM separated the groups according to the functional activity in regions including the PFC, IC, and thalamus • For controlled pain-eliciting stimulus on forearm, SVM separated the groups according to functional activity in brain regions including dlPFC, rostral ventral premotor cortex, and inferior parietal lobule
**ASL and MRS (*n* = 3)**									
Gerstner et al. [58]	MRS	Metabolite levels	Myofascial TMD (RDC/TMD)	1M, 10F; 25.8 ± 2.33 y	6 months to 7 years	NA^b^	SF-MPQ [33], STPI [34]	1M, 10F; 24.8 ± 1.2 y	• Glu levels lower in all individuals after pain testing • In TMD: - Left-insular Gln levels were related to reported pain - Left posterior insular NAA and Cho levels higher at baseline - Left insular NAA levels positively correlated with pain symptom duration
Youssef et al. [23]	ASL	CBF and brain stem blood flow	TMD (RDC/TMD)	3M, 12F; mean ± SEM, 44.9 ± 3.1 (25–67) y	11.4 ± 3.3 y	5	VAS^2^, MPQ [36]	13M, 41F; mean ± SEM, 46.9 ± 2.1 (20–80) y	• TNP had CBF decreases in several regions, including thalamus, S1 and cerebellar cortices • TMD had CBF increases in regions associated with higher-order cognitive and emotional functions, such as ACC, dlPFC and precuneus • In TMD, blood flow increased in motor-related regions and within spinal trigeminal nucleus
Harfeldt et al. [59]	MRS	Metabolite levels	rTMD and gTMD (DC/TMD)	rTMD: 17F, median age, 40 (30–44) y; gTMD: 19F, median age, 43 (40–56) y	≥3 months	NA	NRS^3^	10F; median age, 36 (26–51) y	• Only tCr level was higher in TMD than HC • Cho negatively correlated to maximum mouth opening capacity with or without pain, as well as PPT at the hand • Glu positively correlated to temporal summation and the rTMD and gTMD pain groups showed more pronounced temporal summation • gTMD pain group had lower PPT than rTMD

^a^Age and disease duration give as mean ± SD (range), unless stated otherwise (e.g. mean ± SEM, median)

^b^The study did not report the details of individual medication status but asked patients to be free of medication before MRI scanning

^1^Numerical pain scale (range 0–10, 0 = “no pain”, 10 = “the worst pain imaginable”)

^2^Visual analogue scale (range 0–10, 0 = “no pain”, 10 = “the worst pain imaginable”)

^3^Numerical rating scale (range 0–10, 0 = “no pain”, 10 = “most possible pain”)

*Abbreviations:n* number; *M* male; *F* female; *SD* standard deviation; *SEM* standard error of the mean; *y* year (s); *NA* not applicable; *VAS* visual analog scale; *SF-MPQ* short-form McGill Pain Questionnaire; *MPQ* McGill Pain Questionnaire; *NRS* numerical rating scale; *NPS* numeric pain scale; *SCL-90* Symptom Check List-90; *Di* dysfunction index; *GCPS* Graded Chronic Pain Scale; *TMD* temporomandibular disorders; *RDC/TMD* (diagnosed using) Research Diagnostic Criteria for TMD; *DC/TMD* (diagnosed using) Diagnostic Criteria for TMD; *HC* healthy controls; *MRI* magnetic resonance imaging; *fMRI* functional MRI; *MRS* magnetic resonance spectroscopy; *rs-fMRI* resting-state fMRI; *ts-fMRI* task-state fMRI; *ASL* arterial spin labeling; *mPFC* medial prefrontal cortex; *DMN* default mode network; *PCu* precuneus; *PAG* periaqueductal gray; *fALFF* fractional amplitude of low-frequency fluctuation; *SMA* supplementary motor areas; *SVM* support vector machine; *OFC* orbitofrontal cortex; *CR-MI* centric relation-maximum intercuspation; *ACC* anterior cingulate cortex; *FC* functional connectivity; *IC* insular cortex; *Di* disable index; *S1* primary somatosensory cortex; *S2* secondary somatosensory cortex; *pgACC* pregenual ACC; *M1* primary motor cortex; *PCC* posterior cingulate cortex; *PTN* painful trigeminal neuropathy; *FA* fractional anisotropy; *BOLD signal* blood-oxygen-level-dependent signal; *TMJ* temporomandibular joint; *PFC* prefrontal cortex; *dlPFC* dorsolateral PFC; *Gln* Glutamine; *NAA* N-acetyl aspartate; *Cho* choline; *CBF* cerebral blood flow; *tCr* total creatine; *PPT* pressure-pain threshold; *rTMD* regional TMD pain; *gTMD* generalized pain including TMD pain

**Table 2:** Summary of structural MRI studies on TMD, MRI techniques including sMRI and DTI

Studies (***n*** = 9)	Modality	Analysis method	Patient					Controls	Main findings in TMD patients compared to HC
			Characteristics	Number and age*	Duration*	Medication (n)	Clinical assessments	Number and age*	
Younger et al. [31]	3D T1	Whole brain VBM	Myofascial TMD (RDC/TMD)	14F; 38 ± 13.7 (23–61) y	4.4 ± 2.9 (1–11) y	9	NRS^3^	15F, individually age-matched to patients	• No overall difference in GMV between TMD and HC • In TMD: - Decreased or increased GMV in several areas of trigemino-thalamo-cortical pathway, including brainstem trigeminal sensory nuclei, thalamus and S1 - Increased GMV in limbic regions such as posterior putamen, globus pallidus, and anterior IC - Self-reported pain severity was associated with increased GMV in pgACC and PCC
Gerstner et al. [32]	3D T1	Whole brain VBM	Myofascial TMD (RDC/TMD)	9F; 25.4 ± 2.5 (23–31) y	2.5 ± 2.1 (0.5–4) y	NA**	VAS^2^, SF-MPQ [33], STPI [34]	9F; 24.8 ± 1.4 (24–27) y	• No differences in global GMV or WMV • In TMD: - Decreased GMV in left ACC, right PCC, right anterior IC, left inferior frontal gyrus, and superior temporal gyrus - Decreased regional WMV in medial prefrontal cortex bilaterally
Gustin et al. [35]	3D T1 and MRS	Whole brain VBM and metabolite levels	TMD (RDC/TMD)	4M, 16F; mean ± SEM, 45.7 ± 2.9 (28–70) y	11.4 ± 3.3 y	14	VAS^2^, MPQ [36], BDI [37], STATE [38]	6M, 25F; mean ± SEM, 46.8 ± 3.3 (21–87) y	• VBM revealed no change in regional GMV in TMD compared to HC, while TNP had significant regional GMV changes in a number of brain regions • No significant change in NAA/Cr in thalami of TMD compared with HC, while NAA/Cr was decreased in the thalamus in TNP • Regional GMV and thalamic NAA/Cr was negatively correlated to diary pain scores in TNP but not TMD
Moayedi et al. [39]	3D T1	CTA and VBM (ROI)	TMD (RDC/TMD)	17F; 33.1 ± 11.9 y	9.8 ± 8.25 (0.75–30) y	11	NPS^1^, NEO-FFI [40]	17F; 32.2 ± 10.1 (20–50) y	• TMD patients had cortical thickening in S1 and PFC • TMD clinical characteristics were related to brain structure: - GMV in sensory thalamus positively correlated to TMD duration - Cortical thickness in M1 and aMCC negatively correlated to pain intensity - Pain unpleasantness negatively correlated to cortical thickness in OFC - Positive correlation between neuroticism and OFC thickness
Moayedi et al. [41]	3D T1	CTA and VBM (ROI)	Ditto	Ditto	Ditto	Ditto	NPS^1^	Ditto	• TMD had accelerated whole-brain GMV loss compared to HC, but TMD duration was not correlated to GMV • Three types of aberrant relationships between GM and age in five focal brain regions: - TMD had age-related GMV increases in thalamus whereas GM in HC was relatively sustained - TMD had age-related cortical thinning in aMCC/pgACC, while HC had age-related cortical thickening - TMD patients maintained cortical thickness in dorsal striatum and PMC with age, as opposed to age-related GMV decrease in HC. • TMD duration was related to cortical thinning in PMC
Moayedi et al. [42]	DTI	FA, MD, and RD (ROI)	Ditto	Ditto	Ditto	Ditto	NPS^1^	Ditto	In TMD: • Decreased FA in right and left trigeminal nerves, and FA in right trigeminal nerve negatively correlated with TMD duration • Widespread microstructure alterations of WM tracts related to sensory, motor, cognitive and pain functions, including a focal area of the corpus callosum • Corpus callosum had higher connection probability to frontal pole and lower connection probability to dlPFC • FA correlated with TMD clinical characteristics - FA in tracts adjacent to vlPFC and tracts coursing through thalamus negatively correlated with pain intensity - FA in internal capsule negatively correlated with pain intensity and unpleasantness
Salomons et al. [43]	3D T1 and DTI	CTA (ROI) and FA (TBSS)	Ditto	Ditto	Ditto	Ditto	NPS^1^, Pain Catastrophizing Scale [44]	Ditto	In TMD: • Magnitude of self-reported helplessness correlated with cortical thickness in SMA and MCC, regions implicated in cognitive aspects of motor behavior • FA of connected white matter tracts along corticospinal tract was associated with helplessness and mediated the relationship between SMA cortical thickness and helplessness
Wilcox et al. [45]	3D T1 and DTI	VBM (ROI), FA and MD	TMD (RDC/TMD)	4M, 16F; mean ± SEM, 45.7 ± 2.9 (20–78) y	mean ± SEM: 9.15 ± 8.78 (1.5–30) y	14	VAS^2^, MPQ [36]	5M, 21F; mean ± SEM, 52.3 ± 2.95 y	• Trigeminal neuralgia displayed 47% decrease in trigeminal nerve root volume but no change in DTI values • TNP had 40% increase in nerve volume but no change in DTI values • TMD had no change in volume or DTI values
Wilcox et al. [22]	3D T1 and DTI	VBM (ROI), FA and MD	TMD (RDC/TMD)	4M, 18F; mean ± SEM, 46.5 ± 2.6y	median: 9.7 y	15	VAS^2^, MPQ [36]	7M, 33F; mean ± SEM, 48.3 ± 2.1 y	In TMD: • Regional GMV decrease in medullary dorsal horn, in conjunction with an increase in MD • Volumetric and MD changes in regions of the descending pain modulation system, including the PAG and nucleus raphe magnus • Decreased FA in root entry zone of trigeminal nerve, spinal trigeminal tract and ventral trigemino-thalamic tracts

*Age and disease duration represented as mean ± SD (range), unless stated otherwise (e.g. mean ± SEM, median)

**The study did not report the details of individual medication status but asked patients to be free of medication before MRI scanning

^1^Numerical pain scale (range 0–10, 0 = “no pain”, 10 = “the worst pain imaginable”)

^2^Visual analogue scale (range 0–10, 0 = “no pain”, 10 = “the worst pain imaginable”)

^3^Numerical rating scale (range 0–11, 0 = “no pain”, 11 = “most possible pain”)

***Abbreviations:****n* number; *M* male; *F* female; *SD* standard deviation; *SEM* standard error of the mean; *y* years; *NA* not applicable; *VAS* visual analog scale; *SF-MPQ* short-form McGill Pain Questionnaire; *MPQ* McGill Pain Questionnaire; *BDI* Beck Depression Inventory; *STATE* State Anxiety Index; *NRS* numerical rating scale; *NPS* numeric pain scale; *TMD* temporomandibular disorders; *RDC/TMD* (diagnosed using) Research Diagnostic Criteria for TMD; *HC* healthy controls; *MRI* magnetic resonance imaging; *sMRI* structural MRI; *DTI* diffusion tensor imaging; *MRS* magnetic resonance spectroscopy; *GM* gray matter; *GMV* gray matter volume; *WM* white matter; *WMV* white matter volume; *ACC* anterior cingulate cortex; *pgACC* pregenual ACC; *PCC* posterior cingulate cortex; *IC* cingulate cortex; *VBM* voxel-based morphometry; *NAA* N-acetyl aspartate; *NEO* Neuroticism-Extraversion-Openness Five Factor Inventory; *Cr* creatine; *CTA* cortical thickness analysis; *TNP* trigeminal neuropathic pain; *S1* primary somatosensory cortex; *PFC* prefrontal cortex; *M1* primary motor cortex; *aMCC* anterior mid cingulate cortex; *OFC* orbitofrontal cortex; *PMC* premotor cortex; *FA* fractional anisotropy; *MD* mean diffusivity; *RD* radial diffusivity; *dlPFC* dorsolateral PFC; *vlPFC* ventrolateral PFC; *SMA* supplementary motor areas; *PAG* periaqueductal gray

#### Neuroimaging methods employed in the reviewed studies

Of the 25 studies, 4 studies [31, 32, 39, 41] used the single modality of structural MRI (sMRI) to assess GMV changes by voxel-based morphology and/or cortical thickness alterations using surface-based morphometry; of these, 2 studies [31, 32] performed a whole-brain analysis and 2 studies [39, 41] used a region of interest (ROI) approach. Three studies [22, 43, 45] combined sMRI and diffusion tensor imaging (DTI) to explore both GMV and white matter microstructure. One study [42] employed the single modality of DTI to investigate changes of fractional anisotropy (FA) and mean diffusivity (MD) in patients with TMD. Four studies [46–49] used resting-state fMRI (rs-fMRI) to evaluate spontaneous neural function and functional connectivity (FC). Nine studies [50–57, 60] used task-state fMRI (ts-fMRI) to investigate regional changes in neural activity during pain perception and modulation, of which 1 study [51] combined resting and task state fMRI and 1 study [50] mixed 3 modalities of ts-fMRI, DTI, and arterial spin labeling (ASL). One study [23] used the single modality of ASL to measure CBF in the whole brain and the brainstem in two steps. Three studies [35, 58, 59] used proton magnetic resonance spectroscopy (^1^H MRS) to identify the metabolites in the brain of TMD patients; of these 2 studies [58, 59] focused on the posterior insular and 1 study [35] used both sMRI and ^1^H MRS.

#### Characteristics of the subject populations

Notably, there was some overlap in the patient cohorts. Six studies from the University of Toronto recruited the same subjects (17 TMD patients and 17 healthy controls) [39, 41–43, 46, 54]. The patients in 3 research groups of studies partially overlapped: first, 5 studies from the University of Sydney [22, 23, 35, 45, 50]; second, 4 studies from the University of Michigan [32, 51, 56, 58]; third, 3 studies from Sichuan University [47, 48, 57].

Most studies include either only female subjects [31, 32, 39, 41–43, 46, 49, 51, 52, 54, 59] or many more females than males [22, 23, 35, 45, 50, 53, 56, 58, 60]. While some studies specifically focused on patients in a narrow age-range (for example, from 20s to 30s) [32, 47, 48, 51, 52, 56–59], others included a broader range of ages, ranging from 20s to 50s [22, 23, 31, 35, 39, 41–43, 45, 46, 49, 50, 53–55, 60]. Five studies [31, 32, 51, 56, 58] included patients with myofascial TMD and 2 studies [49, 53] investigated patients with TMJ synovitis pain, whereas the other studies included patients with either muscle pain or synovitis pain or both. The average duration of symptoms across the studies ranged from 14 months to 12 years. Pain intensity and other pain-related characteristics were recorded using the visual analog scale [32, 35, 49–51, 53, 55, 56], numeric pain scale [39, 41–43, 46], numerical rating scale [31, 54, 59], McGill pain questionnaire [22, 23, 32, 35, 45, 50–52, 56, 58] or Graded Chronic Pain Scale [47, 48, 55, 57, 60].

Four studies [47–49, 57] reported that patients took no medication for TMD treatment before. Five studies [52, 53, 55, 59, 60] did not report whether patients were medicated for TMD or not. Other studies reported details on individual medication status and/or asked patients to be free of pain medications before MRI scanning [22, 23, 31, 32, 35, 39, 41–43, 45, 46, 50, 51, 54, 56, 58].

#### Quality assessment

Quality scores for each study are reported in Additional file 1: Table S1. A total score of 9 was possible. For the study not including a control group [60], a maximum score of 7 was allowed. The majority of included studies showed a moderate to high score of methodological quality assessment. A prevalent strength of included studies was the detailed descriptions of the patient and control groups, in which the age and sex were matched, although a few studies [23, 35, 45, 50, 52, 60] did not report whether recruited TMD patients are free of fibromyalgia or other chronic pain disorders. All studies, except 1 with 2.0 Tesla [53], used 3.0 Tesla MRI scanner.

### Alterations in the trigemino-thalamo-cortical pathway

#### Alterations in the trigeminal nerve roots

In MRI studies, patients with TMD displayed significantly lower FA [22, 42], higher MD [22] and decreased GMV [22] in the trigeminal nerve root compared with healthy controls. A negative correlation was found between the FA in the right trigeminal nerve root and TMD duration [42]. However, an earlier TMD study [45] found no diffusivity changes in the trigeminal nerve root using a manual ROI analysis.

#### Alterations in the brainstem

Reports of volumetric changes in the trigeminal principal sensory nucleus (Vp) in TMD are inconsistent. Wilcox et al. [22] found decreased GMV in the ipsilateral subnucleus interpolaris and caudalis of the spinal trigeminal nucleus as well as the ipsilateral Vp in TMD patients, which was the opposite of an increased GMV in Vp reported by Younger et al. [31]. Wilcox et al. [22] suggested that the discrepancy may arise from the differences in pain duration between the patients (4.4 vs 9.7 years), possibly reflecting short-term compensatory volume increase followed by volume decrease over a longer-term course of illness [22]. In addition, another MRI study [23] using ASL detected a significant increase of blood flow in several brainstem regions in TMD patients, including the right SpVc, right Vp, and rostral pons encompassing the ventral trigemino-thalamic tract.

Wilcox et al. [22] also used DTI to investigate microstructural alterations in the brainstem, focusing on the SpVc and pain-processing pathways. Compared with 40 pain-free controls, 22 patients with TMD showed a significant increase of MD in the ipsilateral spinal trigeminal nucleus, bilateral trigeminal nerve tract within the pons and PAG [22]. Another DTI study [42] confirmed aberrant peripheral input from the trigeminal nerve, finding lower FA in the brainstem white matter along the ascending nociceptive pathways coursing through the thalamus, internal capsule and tracts projecting to sensorimotor cortex.

#### Alterations in the thalamus and S1

The thalamus and S1 are chief projections from the trigeminal nerve system [61] and play essential roles in the thalamocortical pathway related to pain [62]. The ventral posterior thalamus receives nociceptive and other sensory information from the periphery and projects to S1 [63]. Two studies [31, 39] reported significantly increased GMV of thalamus in TMD compared to controls, and 1 found a positive correlation between the thalamus GMV and TMD duration [39]. Additionally, age-related GMV increase in the thalamus was found in TMD patients [41], compared to a weak age-related GMV decrease in healthy controls.

Anatomical MRI studies of S1 changes in TMD have generated less consistent results, with different studies reporting decreased GMV [31], increased cortical thickness [39] or no change in GMV [35]. This divergence may result from differences in pain duration, as well as the impact of medication. To investigate changes of S1 in TMD further, Gustin et al. [50] used multiple modalities of MRI (ts-fMRI, DTI, and ASL) to determine whether S1 reorganization occurred in both neuropathic (e.g. painful trigeminal neuropathy [PTN]) and non-neuropathic (e.g. TMD) pain. They found that innocuous brushing of the lip, thumb and little finger resulted in similar functional activation in S1 of TMD patients and controls, and found no significant differences in FA or CBF within contralateral S1 between TMD patients and controls, while PTN patients displayed functional reorganization evidenced by reduced FA and CBF in S1 [50]. Another ASL study [23] found no significant CBF changes in the thalamus and S1 in patients with TMD, as opposed to CBF decrease in the 2 areas in PTN patients. Gustin et al. [50] suggested that the critical factor for S1 reorganization may be the constant S1 input, regardless of pain type (i.e. neuropathic vs non-neuropathic pain); the lack of increased S1 activity in TMD patients as revealed by ASL might reflect a lack of constant nociceptive input to S1 as a general feature of TMD patients [50].

### Alterations in cortical regions

#### Altered lateral and medial pain systems

The lateral and medial pain systems are involved in the nociceptive processing of pain and responsible for individual differences in its experience. The lateral pain system mainly carries information to the lateral thalamic nuclei which projects to S1, secondary somatosensory cortex, posterior insula and mid-cingulate cortex (MCC), etc., and is believed to encode pain intensity, laterality and somatotopy [64]. The medial pain system is implicated in mediating the more affective-motivational aspects in the experience of pain. This circuitry mainly relays information through the medial thalamic nuclei to the anterior insula and anterior cingulate cortex (ACC), integrating interoceptive input with its emotional salience [65].

The insula and cingulate cortex are the most consistently activated forebrain regions when someone experiences pain. Younger et al. [31], studying 15 female patients with myofascial TMD, first reported increased GMV in the right anterior insula and a negative correlation between self-reported pain intensity and GMV in the pregenual ACC (pgACC) and posterior cingulate cortex (PCC). Ichesco et al. [51] investigated FC between insula and cingulate cortex in 8 female patients with TMD and found elevated FC between left anterior insula and pgACC in resting state. They also showed that during a task state with an applied pressure pain as a controlled stimulus, this FC was negatively correlated with subjective pain intensity [51], i.e. patients with increased connectivity reported lower pain, suggesting compensatory brain changes to regulate pain in patients. A recent rs-fMRI study by Zhang et al. [49] found decreased regional homogeneity in the right anterior insula in 8 female patients with TMJ synovitis pain relative to 10 healthy controls. Gerstner et al. [32] reported decreased GMV in the right anterior insula, ACC, and PCC in 9 female TMD patients, suggesting that via excessive stimulation or increased compensatory inhibitory input, anatomic changes may also be observed.

In regard to MCC involved in the lateral pain system, Zhang et al. [49] reported decreased FC between MCC and anterior insula, which was negatively correlated with pain intensity in TMD patients, i.e. patients with decreased connectivity reported higher pain. MRI studies investigating TMD patients have also reported decreased FC between MCC and dorsolateral prefrontal cortex (dlPFC) [54], lower FA in MCC [42] and a negative correlation between the cortical thickness of MCC and pain intensity [39].

The involvement of posterior insula in TMD-related chronic pain has also been noted. Two ^1^H MRS studies [58, 59] investigated neurochemistry in the posterior insula of TMD patients. MRS-detectable neurometabolites include glutamate (Glu), a major excitatory neurotransmitter contributing to the negative affect associated with pain [66], and its metabolite glutamine (Gln), both involved in complex metabolic cycles between neurons and astrocytes [67]. MRS studies have also examined N-acetyl aspartate (NAA), choline (Cho) and total creatine (tCr), markers of other aspects of neurochemistry. Gerstner et al. [58] found a negative correlation between Gln levels in the left insula and reported pain in 11 TMD patients; NAA and Cho levels in the left posterior insula were increased compared to 11 healthy controls and NAA levels were positively correlated with the duration of pain. In addition, Harfeldt et al. [59] reported elevated tCr levels within the posterior insula in 17 TMD patients relative to 10 healthy controls; in the patient group, increased Cho levels correlated with a reduced capacity for mouth opening and lower pressure pain threshold on the hand, while Glu levels were positively correlated with temporal summation of the nociceptive mechanical stimulus.

#### Altered DMN and pain modulation

The DMN is a group of functionally interconnected brain regions known to be active when people are mind wandering and not involved in any specific task, which becomes correspondingly deactivated during goal-oriented tasks [68]. Activity in this network reflects self-monitoring activity and the processing of internal state information. In patients with TMD, Weissman-Fogel et al. [54] reported task-evoked activation in the medial prefrontal cortex (mPFC) and PCC, as well as functional dysconnections within the DMN in TMD patients performing a task with emotional interference. In addition, Kucyi et al. [46] found enhanced FC between mPFC and other DMN regions including PCC/precuneus (PCu), retrosplenial cortex, and areas within visual cortex. Furthermore, the pain rumination scores were positively correlated with the FC between mPFC and PCC/PCu, retrosplenial cortex, mediodorsal thalamus, and PAG [46].

The PAG-raphe magnus system is the best-studied pathway for descending pain modulation [69]. Wilcox et al. [22] demonstrated that in TMD subjects, the PAG displayed a significant increase of MD value and no GMV change, while the nucleus raphe magnus showed GMV decrease and no change in diffusivity.

A number of higher-level brain areas have also proved to be engaged in pain control, including the cingulo-frontal regions, amygdala, and hypothalamus [70]. Increased CBF has been found in TMD patients in brain regions implicated in cognitive and emotional functions, including the ACC, dlPFC and PCu [23]. Patients with TMD often show impaired cognitive ability on neuropsychological testing [71, 72]. Weissman-Fogel et al. [54] demonstrated that TMD patients had aberrant brain responses while performing the attention-demanding Stroop task with cognitive and emotional interference, including reduced FC within two pairs of brain regions, the anterior MCC [aMCC]-dlPFC and pgACC-amygdala. Another study [39] employing sMRI reported that TMD patients had thicker cortex in the frontal pole and ventrolateral prefrontal cortex compared with healthy controls, and that cortical thickness of orbitofrontal cortex (OFC) was negatively correlated to pain unpleasantness. Additionally, cortical thickness in the left ventromedial prefrontal cortex (vmPFC, part of OFC) was positively correlated with neuroticism scores [40] in TMD patients, different from a negative correlation in healthy subjects [73].

### Alterations in the motor system

Though this is less well-explored than the sensory-discriminative and affective-motivational components of pain, there is increasing evidence supporting involvement of the motor system in patients with TMD-related pain. Wessman-Fogel et al. [54] found that TMD patients showed elevated activity in the primary motor cortex (M1) and supplementary motor areas (SMA) during the cognitive interference Stroop task. He et al. [47] identified decreased neural spontaneous function in M1 and SMA in treatment-naive TMD patients, measured by the fractional amplitude of low-frequency fluctuation (fALFF) calculated from rs-fMRI data. Centric relation-maximum intercuspation (CR-MI) discrepancy of bilateral TMJs is an indicator of the presence and severity of TMD [74, 75], and He et al. [47] found a negative correlation between the fALFF in M1 and vertical CR-MI [76] in TMD patients. Furthermore, in an sMRI study by Salomons et al., [43] self-reported helplessness in TMD patients assessed by Pain Catastrophizing Scale [44] was positively correlated with the cortical thickness of SMA, a critical region implicated in cognitive aspects of motor behavior [77], but further analysis [43] identified neither significant group difference in the cortical thickness of SMA nor correlation with pain characteristics.

In addition to M1 and SMA, the striatum has also been implicated in the motor response to pain in TMD. TMD patients with myofascial pain had increased GMV in the right putamen and right globus pallidus relative to controls [31]. TMD patients also showed sustained increased GMV in the dorsal striatum independent of TMD duration, while healthy controls had normal age-related gray matter atrophy in this region [41], suggesting an aberrant pattern of striatal aging in TMD. In addition, a rs-fMRI [48] study explored the FC of the corticostriatal circuit in patients with TMD. Compared with controls, the patient group had reduced FC in the ventral corticostriatal circuitry between the ventral striatum and ventral-frontal cortices (ACC and anterior insula), in the dorsal corticostriatal circuitry between dorsal striatum and dorsal cortices (precentral gyrus and supramarginal gyrus), and also within the striatum [48]. Exploratory analysis found associations between decreased corticostriatal FC and clinical variables of overall clinical dysfunction measured by Helkimo indices [78] and pain intensity [48].

### Altered brain activations in response to mechanical stimuli

Previous studies found that patients with TMD had abnormal vibrotactile sensibility on the face, with elevated detection threshold [79] and impaired frequency discrimination [80], indicating a disruption of the somatosensory system in TMD. Compared with healthy controls, with the stimulation of low frequency and innocuous vibration of the index finger, patients with TMD displayed greater activations in bilateral ACC and contralateral amygdala [52], 2 critical regions implicated in the emotional aspect of pain [81, 82]. In addition, 16 TMD synovitis patients with biting pain showed elevated activation of ACC during clenching tasks compared with 14 controls, and the activation of ACC was found to be associated with higher levels of psychological distress in patients [53].

Multivariate analysis techniques have begun to be used to identify the central mechanisms underlying pain processing in TMD. Roy et al. [55] investigated neural activity during a grip-force task and a pain-eliciting forearm thermal stimulus in TMD patients with chronic jaw pain [83] and controls, using multivariate analyses to identify brain regions whose functional activity could discriminate between the groups. By using multivariate analyses, in the motor control task, increased activity in brain areas including PFC, insula, and thalamus could distinguish the patients from controls with a mean test for the area under the receiver operator characteristic curve (AUC) of 0.8, although the parameters of grip-force production were similar in both groups [55]. In the pain task, there was no significant difference in stimulus intensity and pain perception between groups, but the functional activations of dlPFC, ventral premotor cortex, and inferior parietal lobule could distinguish the groups with a mean test AUC of 0.85 [55].

Harper et al. [56] explored the differences in brain activity evoked by subjectively equated pain originated from temporalis (a clinically painful region) and thumb (a remote asymptomatic area) in patients with TMD and controls. The support vector machine (SVM) method was able to distinguish between the neural activations in areas with cognitive valuation of pain, including the left OFC, ACC, and operculum in response to temporalis-evoked pain and thumb-evoked pain in TMD patients with an accuracy of 75%, but not in healthy controls (55%) [56].

### Brain changes after splint therapy

Lickteig et al. [84] first used fMRI to record cerebral activation changes of a single TMD patient before and after splint therapy (individually optimized, applied for 11 nights and partial days) and found decreased brain activity (in bilateral sensorimotor regions and other areas such as left posterior insula) during occlusion (both on natural teeth and splint) after treatment compared to the untreated baseline. The same group performed a longitudinal fMRI study to assess the effect of 2-week splint therapy on 14 patients with TMD [60]. After splint treatment the patients showed reduced activations in the right anterior insula and right cerebellar hemisphere during occlusion, accompanied by relief of subjective pain and increased symmetry of condylar movements. Correlation analysis identified an association between reduced pain scores and attenuated activations in the right anterior insula, left posterior insula and left cerebellar hemisphere, as well as between improved condylar movements and decreased activations in the left cerebellar and right M1 [60].

He et al. [47] used rs-fMRI to investigate the spontaneous brain activity of 23 TMD patients with CR-MI discrepancy and assessed the therapeutic effect of maxillary stabilization splint. Eleven out of 23 patients received 3 months splint therapy, showing improved signs and symptoms and more stable condylar position; the fALFF in left M1 and left posterior insula, which had been decreased before treatment compared to healthy controls, recovered to the normal level [47]. Another study [57] explored neural activation in TMD patients during a clenching task before and after stabilization splint therapy. Compared with the control group, TMD patients before treatment exhibited decreased positive activations in cerebral areas associated with motor and cognitive functions (including the left M1, bilateral inferior temporal gyrus and left cerebellum) and elevated negative activations in the DMN (right mPFC). Neural activity in these cortical regions normalized after splint therapy, accompanied by improvement of TMJ status [47].

## Discussion

**Fig. 2:**
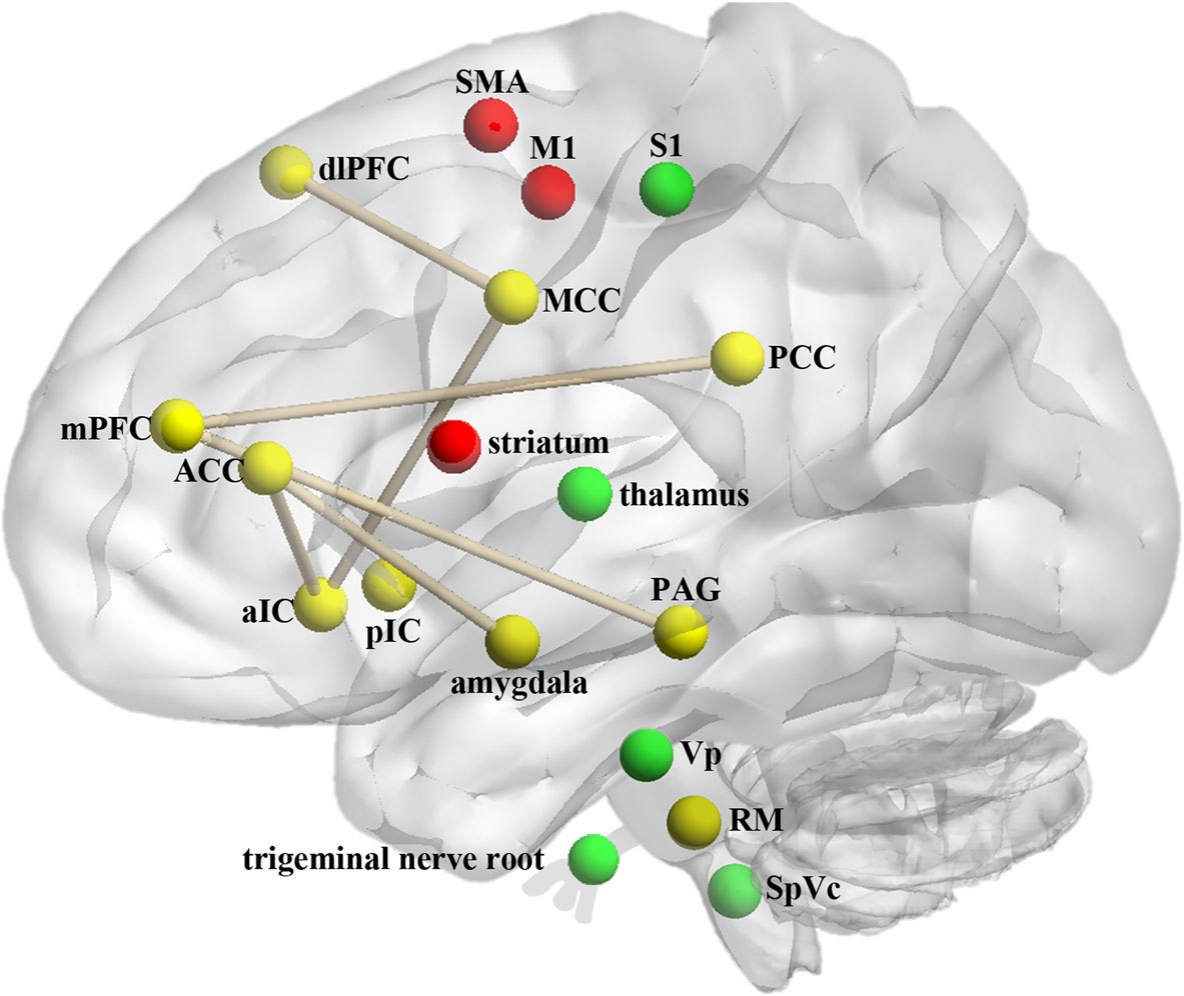
Schematic representation of main brain regions with altered structure and function involved in TMD related-pain. Green balls represent the areas in the classic trigemino-thalamo-cortical system. Red balls are in the motor system. Yellow balls are the brain cortical regions implicated in pain perception and pain modulation. Brain regions with altered functional connectivity in TMD are connected with lines in khaki. ***Abbreviations:*** SMA, supplementary motor areas; dlPFC, dorsolateral prefrontal cortex; M1, primary motor cortex; S1, primary somatosensory cortex; MCC, mid-cingulate cortex; mPFC, medial prefrontal cortex; ACC, anterior cingulate cortex; PCC, posterior cingulate cortex; aIC, anterior insular cortex; pIC, posterior insular cortex; PAG, periaqueductal gray; Vp, trigeminal principal sensory nucleus; RM, raphe magnus; SpVc, spinal tract subnucleus caudalis

In this review, 25 original MRI studies were retrieved to investigate the neuro-pathophysiological manifestations of TMD-related pain. Notably, our findings provide evidence for both peripheral and central neural basis for pain in TMD (briefly summarized in Fig. 2). The lack of large sample size studies and the variety of methodological approaches complicate the process of synthesizing findings to reach broad conclusions and may explain some discrepancies in results between studies. The following discussion will provide a detailed interpretation of the findings and their clinical implications in a broader context.

### Implications of findings in the trigemino-thalamo-cortical pathway

The classic trigemino-thalamo-cortical pathway is responsible for the sensation of oral and maxillofacial regions [85]. Current evidence reveals brain structural and functional alteration in key nodes within this ascending trigemino-thalamo-cortical pathway in TMD.

Diffusivity changes observed in the trigeminal nerve root [22, 42] may reflect long-term microstructural alterations of the nerve, a manifestation of microstructural changes in response to increased peripheral nociceptive input. The reported discrepancy of diffusivity changes in the trigeminal nerve [22, 45] results from methodological differences between the two studies (with the same samples). The earlier study [45] used manual ROI analysis and the results could be affected by partial volume effects given the small size of the trigeminal nerve root, while the deterministic tractography used in the later study [22] provided greater sensitivity and a more accurate estimate of diffusion properties of white matter tracts [86]. Additionally, the GMV decrease and MD increase in SpVc [22] may reflect glia shrinkage/atrophy or neuronal loss [87] and a reduction in dendritic density [88], while the elevated blood flow in SpVc [35] could be a compensatory response of increased neural activity to structural reductions. As such, the neural structural and blood flow changes in the initial part of the trigeminal system (the root entry zone and SpVc) indicate that the established hyperexcitability of the nociceptive processing pathways is critical for the altered perception and maintenance of pain in TMD [89]. On the other hand, increased GMV and aberrant age-related GMV changes in the thalamus may also be due to persistent trigeminal nociceptive input, which further contributed to the hyperalgesia of TMD by enhanced facilitating trigeminal sensory information from the thalamus to S1.

However, the structural changes of S1 are somewhat less consistent. Gustin et al. [50] found no functional reorganization in the S1 of TMD patients and no significant difference in FA or CBF within S1 compared with healthy controls. This may not necessarily rule out alterations within the trigemino-thalamo-cortical pathway in the TMD patients examined by Gustin et al. [50], as the authors did not investigate the structural and functional changes of other critical areas within the pathway, especially the SpVc and thalamus. Therefore, further MRI studies focusing simultaneously on the SpVc, thalamus, and S1 and examining both brain anatomy and function are required to resolve the issue of inconsistencies of S1 changes in TMD patients. If structural and functional alterations were found only in SpVc and/or thalamus, but not S1, it would be interesting to investigate how TMD patients deal with increased nociceptive inputs while showing intact S1. Alternatively, more refined measurements and larger sample studies may provide a clearer picture of alterations that may occur in S1 in TMD patients.

### Implications of cortical findings

#### Altered lateral and medial pain systems

The ACC and anterior insula are two important regions responsible for encoding the emotional and motivational aspects of pain. Despite some inconsistencies, current structural and functional findings in these two structures in TMD patients [31, 32, 49, 51, 53] support the role of abnormal medial pain system in nociceptive processing, demonstrating the emotional sensory signals related to pain in TMD patients.

Zhang et al. [49] found decreased FC between MCC and anterior insula in TMD patients, while Ichesco et al. [51] reported increased FC between pgACC and anterior insula. FC between anterior insula and different regions of cingulate cortex may represent different brain functional alterations in different disease severity of TMD, since the former group [49] studied patients with severe open-mouth pain (≥5 on the visual analog scale), while the latter [51] recruited patients who were only mildly affected by TMD. We noted that the reduced FC between MCC and anterior insula and the increased FC between pgACC and anterior insula were both negatively correlated with pain intensity of TMD patients, so that these two alterations in FC may represent maladaptive alterations resulted from pain in TMD or compensatory brain changes for pain regulation, respectively.

The posterior insula of TMD patients showed cellular and molecular changes in 2 neurochemical studies [58, 59]. The positive correlation between the NAA levels in left posterior insula and duration of TMD pain suggested a time-dependent neuronal or axonal proliferation in response to pain [58], as NAA is considered as a biomarker of neuronal health and synaptic integrity [90]. In addition, since tCr can be considered as a biomarker of cell energetics [90], the increased tCr levels in the posterior insula in TMD patients [59] may indicate a state of neuroinflammation or cellular hyperactivity which has been proposed to be implicated in chronic pain [91].

#### Dysfunctional DMN and pain modulation

Previous studies have suggested that dysfunction of the DMN may be related to cognitive and behavioral deficits observed in patients with chronic pain [92]. In TMD patients, task-related activation, rather than typical task-related deactivation in DMN areas including the mPFC and PCC, has been observed [54]. PCC can be activated by emotionally salient stimuli and is involved in contextualizing painful stimuli [93]. The increased activation of PCC may indicate increased spontaneous pain in patients when completing cognitive tasks with emotional interference since it had greater emotional effects on patients than controls. Accordingly, mPFC was recruited during the emotion provoking task to mediate antinociceptive effects relevant to its role in descending pain modulation [94]. Moreover, given that mPFC and PCC are 2 functionally connected regions implicated in attention toward introspective thoughts [95, 96] which is required for coping with pain, their abnormal activation during task may reflect a nocifensive mechanism for patients to relieve pain by focusing on internal thoughts, which in turn leads to slower behavioral responses.

Pain rumination is perseverative negative thinking about pain. Kucyi et al. [46] linked dysfunctional DMN with pain rumination in TMD and suggested that individuals with high pain rumination measured by Pain Catastrophizing Scale [44] had particularly enhanced FC between components of the DMN including mPFC and PCC/PCu. The positive correlation between pain rumination and FC of mPFC with mediodorsal thalamus may reflect patients’ persistent attempts to regulate pain, since mediodorsal thalamus is associated with the affective and emotional aspects of pain [97]. In addition, pain rumination was positively correlated with FC between mPFC and PAG, which may account for differential abilities in pain modulation given the prominent role of PAG in descending pain modulation. Since healthy controls had no such a correlation, Kucyi et al. [46] suggested that the degree to which chronic pain alters the normal function of these circuits may depend on how much patients ruminate, supporting the critical role of pain-related cognition in TMD-related brain alterations.

As a key region of the endogenous pain inhibitory system, PAG is well-positioned to modulate pain perception for interactions between ascending inputs from peripheral tissue and descending projections from brain regions (e.g., ACC and mPFC), and shows anatomical alterations in other chronic pain conditions [98, 99]. Since nucleus raphe magnus serves as the recipient in the descending analgesic pathway of PAG [100], reduced dendritic spine numbers in the PAG may alter the descending input to raphe magnus, variably impacting the efficiency of endogenous analgesia. Thus, the dysfunction of the PAG-raphe magnus descending modulatory pathway may shed light on the mechanism underpinning the central sensitization to pain in TMD patients.

It has been established that pain can induce attentional biases [101], and attentional manipulation is able to modulate the perception of pain [102–104]. This highlights the importance of studying brain attentional systems in relation to pain in TMD. In the study by Weissman-Fogel et al. [54], TMD patients showed slower task responses, with reduced FC within two pairs of brain regions (aMCC-dlPFC and pgACC-amygdala). The amygdala is critically implicated in the experience and expression of emotion [81, 82], and pgACC can modulate the reactivity of amygdala when confronted with emotionally salient stimuli [105, 106]. aMCC and dlPFC are highly functionally connected; the activation of aMCC can capture attention to adjust behavior [107, 108], which is mediated by a top-down process that engages dlPFC. Therefore, reduced connections between these two pairs of structures in TMD patients may suggest an influence of their chronic pain onto attentional and emotion networks, which results in attenuated and unsynchronized recruitment of attention-processing areas and consequently slower behavioral responses.

Neuroticism is one of the ‘Big Five’ personality traits and individuals with high neuroticism are more likely to interpret common situations as threatening and minor frustrations as hopeless. It is a personality feature associated with increased levels of current and future anxiety and distress [109, 110]. High neuroticism is also correlated with heightened pain-related suffering [111] and sensitivity [112]. In TMD patients, Moayedi et al. [39] found a positive correlation between cortical thickness in vmPFC and neuroticism relative to a normal negative correlation in healthy controls [73]. While neuroticism is generally a stable trait, it also can vary with level of anxiety and distress associated with physical and psychiatric illness. Thus, observations of associations with neuroticism suggest that greater levels of distress and anxiety in TMD are associated with gray matter changes in the vmPFC of TMD patients, which may result from or contribute to reductions in the brain’s capacity for pain control.

### Motor aspects of TMD-related pain

Neuroimaging studies in patients with TMD pain have reported structural and functional changes in the motor system, including M1, SMA, and striatum. Persistent pain can inhibit protective movement and impair motor performance due to maladaptive neuroplasticity in the motor cortex [113, 114]. Increased activity in M1 and SMA during cognitive interference Stroop task in TMD patients [54] may indicate a possible compensatory mechanism to recruit motor areas to meet elevated demands for motor planning and performance.

Uncontrollable stressors like persistent chronic pain can lead to learned helplessness, a maladaptive response featured by reduced motor escape behaviors and deficits in motivation and learning [115]. The SMA has been implicated in motor planning and pain processing when pain is perceived to be uncontrollable [77, 116]. In the study by Salomons et al. [43], the cortical thickness in SMA did not differ between groups or show any significant correlation with chronic pain characteristics, indicating that the positive correlation between cortical thickness and helplessness was not derived from the cumulative effects of pain. Rather, helplessness in patients with painful TMD may be a function of interactions between predisposing factors, i.e. structural characteristics of nervous systems implicated in motor planning and function and persistent exposure to uncontrollable chronic pain.

As the major component of basal ganglia, striatum receives input from cortical regions and thalamic nuclei, and sends output to other structures of basal ganglia, serving as a critical site where cognitive, motor and limbic signals from other brain regions overlap and are integrated [117, 118]. Findings of increased GMV in the right putamen and right globus pallidus [31], which contain neurons responsive to nociceptive stimuli and function for preparing behavioral responses [119], suggest a possible somatotopic reorganization or perhaps synaptic hypertrophy associated with sustained TMD pain. Correlations between reduced corticostriatal FC and clinical measurements [48] highlight the critical role of the striatum and corticostriatal loops in the motor response to pain.

### Altered brain activations in response to mechanical stimuli

Compared with healthy controls, TMD patients display perceptual amplification of pressure stimulation across a wide range of physical intensities, from gentle and innocuous to strongly painful [120]. fMRI allows the detection of differences in brain function in processing external stimuli between TMD patients and healthy subjects. The differences in the location and magnitude of brain activation responsive to innocuous vibrotactile stimulation between patients and healthy controls and the activation of pain-related regions by innocuous input, as reported by Nebel et al. [52], may reflect the dysfunction of the somatosensory system in individuals with TMD. Findings of Harper et al. [56] using SVM provided further support for the involvement of aberrant central pain processing in patients with TMD pain. The ability of SVM to discriminate the location of noxious stimuli only in TMD patients by different functional activity in regions including OFC, ACC, and operculum indicate somatotopic-dependent differences in pain processing, reflecting regional differences of brain activation related to the cognitive evaluation of pain between TMD patients and healthy subjects.

Although TMD, like many chronic pain conditions, is accompanied by motor impairment, few MRI studies have directly investigated neural activity during a motor task in chronic pain. Roy et al. [55] first investigated brain response during a grip force task and a thermal pain stimulus in TMD patients with chronic jaw pain and healthy controls, and identified brain regions with significantly different functional activities which could separate the groups. They found that TMD patients with chronic jaw pain had different brain processing of motor- and pain- related stimulus compared with healthy controls, which supports the idea that chronic pain is correlated with task-specific brain alterations in the transformation of sensory input to motor activity and to pain perception.

### Neural therapeutic mechanism of splint therapy

Treatment for TMD has been focused on alleviating the main symptoms, especially chronic pain, and the palliative approaches which prevail over surgery have relatively satisfactory clinical outcomes [3, 121, 122]. Splint therapy is one of the commonest conservative treatments for TMD. A recent meta-analysis evaluating various oral orthotic appliances concluded that hard stabilization splints, when adjusted properly, have good evidence of modest efficacy in reducing TMD-related pain relative to non-occluding appliances and no treatment [123]. Stabilization splints are designed to improve the functional movements of TMJs by eliminating occlusal interferences and removing the impact of maximization intercuspation occlusion on joint position, and also improve masticatory function by reducing abnormal muscular activity [124, 125]. It has also been hypothesized that splints can increase patients’ cognitive awareness of oral parafunctional habits, thus altering proprioceptive input and central motor areas implicated in masticatory function [126, 127].

The recent fMRI studies by Lickteig et al. [60, 84] and He et al. [47, 57] revealed the neural mechanisms of splint therapy, finding that it may work partly by eliciting neuroplastic recovery of affected brain regions. Lickteig et al. [60, 84] found decreased activations in brain regions accompanied by improved clinical symptoms after splint treatment compared with pretreatment levels, but there was no control group to assess whether they recovered to normal. On the other hand, He and coworkers [47, 57] found that TMD patients exhibited recovery of previously decreased baseline neural activity after splint therapy, compared with healthy controls. This discrepancy may result from differences in patients’ age, medication usage and splint design. Future MRI studies investigating the therapeutic effects of splint therapy for TMD are expected to identify a general mode of splint impact on the brain of TMD patients.

### Limitations

The present review, and the field of TMD brain imaging research, have several limitations. The first important consideration is the difference in pain origins and TMD patients recruited in studies. Since TMD is an umbrella term encompassing a number of painful conditions involving masticatory muscles, TMJ and associated structures [128], the large variability in disease characteristics of patient groups precludes drawing a definite conclusion about brain changes in TMD with a specific type and pain origin. Studies seeking to identify subgroups of patients in large samples of TMD patients are needed to resolve this important issue.

Another consideration is the gender of participants. The sex ratio of patients seeking medical advice has been reported as ranging from 3:1 (women: men) to as high as 9:1 [129, 130]. Accordingly, there were many more female subjects in the studies discussed in this review. However, no neuroimaging studies of TMD have specifically targeted gender differences or investigated the neural basis of such high morbidity in women.

A third factor that impacts neuroimaging studies of TMD is the potential impacts of analgesics, which may reduce some functional alterations and induce other functional changes that might impact the experience of persistent TMD-related pain. Studying untreated TMD patients at illness presentation, as in other disorders, would be a strategy to advance clarity of illness related alterations.

Fourth, causal relationships are inherently difficult to establish in cross-sectional studies. Among the brain alterations in TMD patients, differentiating features that are the consequence of persistent pain or represent compensation efforts to reduce pain can not be confidently determined at this point, which requires further research. Moreover, findings from existing studies suggest the possibility that certain structural characteristics of the brain associated with levels of anxiety and distress and helplessness may either make some people more vulnerable to developing TMD or represent responses to TMJ-related pain with their own impact on brain anatomy and function.

Last, given that TMD is a multifactorial disease, understanding the personal difference in central sensitization is critical for choosing proper clinical management. Therefore, the combination of neuroimaging techniques and machine learning algorithms such as SVM may serve to benefit future studies of the central mechanisms underlying TMD pain, and eventually facilitate clinical practice by providing new diagnostic strategies, as well as objective measures of therapeutic efficacy.

## Conclusion

This article systematically reviewed the existing literature investigating brain changes in TMD patients for revealing the pathogenic mechanisms or consequences of TMD-related pain. MRI neuroimaging techniques have provided a deeper understanding of what happens to brain structure and function in patients with TMD, suggesting both peripheral and central neural basis for the most common conditions of TMD, i.e. hyperexcitability to external stimuli and disrupted pain perception and pain modulation. This review can enhance our understanding of the pathophysiology underlying symptoms of TMD-related pain as targets for treatment development and planning. Primary novel observations include:

- There are structural and functional changes in the classic trigemino-thalamo-cortical system, including peripheral trigeminal nerve roots, brainstem (SpVc and Vp in particular), thalamus, and S1, which provides support for a peripheral origin of TMD. Specifically, the results of increased GMV in the thalamus were consistent, while whether there are alterations in S1 in TMD patients remains to be clarified.
- There are alterations in several cortical regions implicated in pain perception and pain modulation in TMD. Neurochemical alterations are identified in the posterior insula. The altered FCs among anterior insula, pgACC and MCC are correlated with pain intensity. The dysfunctional DMN in TMD patients is characterized by reduced FC in mPFC-PCC/PCu and mPFC-PAG. TMD related pain-attention interaction is mediated by reduced FC in aMCC-dlPFC and pgACC-amygdala. Structural changes in the PAG-raphe magnus pathway may impair the efficiency of the endogenous pain inhibitory system of TMD patients.
- Regional functional brain changes in M1 and SMA, as well as increased GMV and decreased FC in the striatum, indicate the compensatory changes or maladaptive neuroplasticity of the motor system in patients with TMD pain.
- TMD patients displayed different brain activations in the fronto-insulo-thalamo-parietal network under both innocuous and painful stimulus compared with healthy controls, reflecting the involvement of aberrant central pain processing in TMD. Multivariate analysis techniques like SVM may help distinguish the subtypes of TMD patients, i.e. identify whose pain has a more peripheral or a central etiology, which could be clinically useful for optimal treatment.
- Splint therapy can impact the neural function of brain regions in TMD patients, accompanied by improved clinical symptoms, suggesting a central mechanism underlying the therapeutic effects of splint therapy

## Supplementary information

**Additional file 1: Table S1.** The checklist of quality assessment for included studies in the present systematic review.

**Additional file 2: Table S2.** Regions with significant group differences between patients with TMD and controls reported in functional MRI studies included in the present systematic review. **Table S3.** Regions with significant group differences between patients with TMD and controls reported in structural MRI studies included in the present systematic review.

## Data Availability

All data and articles supporting the conclusions of this systematic review are included within the systematic review.

## Abbreviations

TMD: Temporomandibular disorders
TMJ: Temporomandibular joint
RDC/TMD: Research Diagnostic Criteria for TMD
DC/TMD: Diagnostic Criteria for TMD
MRI: Magnetic resonance imaging
DTI: Diffusion tensor imaging
FA: Fractional anisotropy
MD: Mean diffusivity
fMRI: Functional MRI
ts-fMRI: Task-state fMRI
rs-fMRI: Resting-state fMRI
ASL: Arterial spin labeling
MRS: Magnetic resonance spectroscopy
SpVc: Spinal tract subnucleus caudalis
ACC: Anterior cingulate cortex
mPFC: Medial prefrontal cortex
PAG: Periaqueductal gray
S1: Primary somatosensory cortex
MCC: Mid-cingulate cortex
GMV: Gray matter volume
Vp: Trigeminal principal sensory nucleus
PTN: Painful trigeminal neuropathy
CBF: Cerebral blood flow
FC: Functional connectivity
PCC: Posterior cingulate cortex
M1: Primary motor cortex
pgACC: Pregenual anterior cingulate cortex
SMA: Supplementary motor areas
Glu: Glutamate
Gln: Glutamine
NAA: N-acetyl aspartate
Cho: Choline
tCr: Total creatine
dlPFC: Dorsolateral prefrontal cortex
DMN: Default mode network
aMCC: Anterior mid cingulate cortex
OFC: Orbitofrontal cortex
vmPFC: Ventromedial prefrontal cortex
PCu: Precuneus
CR-MI Discrepancy: Centric relation-maximum intercuspation discrepancy
SVM: Support vector machine
fALFF: Fractional amplitude of low-frequency fluctuation
AUC: Area under the receiver operator characteristic curve
