# Supplementary material for: The neuro-pathophysiology of temporomandibular disorders-related pain: a systematic review of structural and functional MRI studies
Source: J Headache Pain. 2020 Jun 19;21(1):78. doi: 10.1186/s10194-020-01131-4 (PMC7304152; doi:10.1186/s10194-020-01131-4)
Supplement: Supplementary file 1 — Additional file 1: Table S1. The checklist of quality assessment for included studies in the present systematic review. [file 10194_2020_1131_MOESM1_ESM.docx]

**Additional file 1**

**Table S1. The checklist of quality assessment for included studies in the present systematic review.**

| **Study** | **1** | **2** | **3** | **4** | **5** | **6** | **7** | **8** | **9** | **Total** |
| --- | --- | --- | --- | --- | --- | --- | --- | --- | --- | --- |
| Kucyi et al. [46] | 1 | 1 | 1 | 1 | 0.5^c^ | 1 | 1 | 1 | 1 | 8.5 |
| He et al. [47] | 1 | 1 | 1 | 1 | 1 | 1 | 1 | 1 | 1 | 9 |
| He et al. [48] | 1 | 1 | 1 | 1 | 1 | 1 | 1 | 1 | 1 | 9 |
| Zhang et al. [49] | 1 | 1 | 1 | 1 | 1 | 1 | 1 | 1 | 1 | 9 |
| Nebel et al. [52] | 1 | 1 | 1 | 0 | 0 | 1 | 1 | 1 | 1 | 7 |
| Ichesco et al. [51] | 1 | 1 | 1 | 1 | 0.5 | 1 | 1 | 1 | 1 | 8.5 |
| Wessman et al. [54] | 1 | 1 | 1 | 1 | 0.5 | 1 | 1 | 1 | 1 | 8.5 |
| Zhao et al. [53] | 1 | 1 | 0 | 1 | 0 | 0 | 1 | 0 | 1 | 5 |
| Gustin et al. [50] | 1 | 1 | 1 | 0 | 0.5 | 1 | 1 | 1 | 1 | 7.5 |
| Lickteig et al. [60] | 1 | NA^a^ | NA^a^ | 0 | 0 | 1 | 1 | 1 | 1 | 5 |
| He et al. [57] | 1 | 1 | 1 | 1 | 1 | 1 | 1 | 1 | 1 | 9 |
| Harper et al. [56] | 1 | 1 | 1 | 1 | 0.5 | 1 | 1 | NA^b^ | 1 | 7.5^b^ |
| Roy et al. [55] | 1 | 1 | 1 | 1 | 0 | 1 | 1 | 1 | 1 | 8 |
| Youssef et al. [23] | 1 | 1 | 1 | 0 | 0.5 | 1 | 1 | 1 | 1 | 7.5 |
| Gerstner et al. [58] | 1 | 1 | 1 | 1 | 0.5 | 1 | 1 | 0 | 1 | 7.5 |
| Harfeldt et al. [59] | 1 | 1 | 1 | 1 | 0 | 1 | 1 | 0 | 1 | 7 |
| Younger et al. [31] | 1 | 1 | 1 | 1 | 0.5 | 1 | 1 | 1 | 1 | 8.5 |
| Gerstner et al. [32] | 1 | 1 | 1 | 1 | 0.5 | 1 | 1 | 0 | 1 | 7.5 |
| Gustin et al. [35] | 1 | 1 | 1 | 0 | 0.5 | 1 | 1 | 1 | 1 | 7.5 |
| Moayedi et al. [39] | 1 | 1 | 1 | 1 | 0.5 | 1 | 1 | 1 | 1 | 8.5 |
| Moayedi et al. [41] | 1 | 1 | 1 | 1 | 0.5 | 1 | 1 | 1 | 1 | 8.5 |
| Salomons et al. [43] | 1 | 1 | 1 | 1 | 0.5 | 1 | 1 | 1 | 1 | 8.5 |
| Wilcox et al. [45] | 1 | 1 | 1 | 0 | 0.5 | 1 | 1 | 0 | 1 | 7.5 |
| Wilcox et al. [22] | 1 | 1 | 1 | 0 | 0.5 | 1 | 1 | 1 | 1 | 7.5 |
| Moayedi et al. [42] | 1 | 1 | 1 | 1 | 0.5 | 1 | 1 | 1 | 1 | 8.5 |

Items：

1. Were the descriptions of the patient group clear and adequate? 1= yes, 0= no or too less information

2. Were the descriptions of the control group clear and adequate? 1= yes, 0= no or too less information

3. Were the patient group comparable to the control group in terms of age and gender? 1= yes, 0= no

4. Whether all patients were free of comorbidity? (assessing the presence of fibromyalgia or other chronic pain disorder) 1= yes, 0= no or too less information

5. Whether the medication history has been reported? 1= without medication for TMD, 0.5= reported or discontinued before MRI scanning, 0= not reported

6. What is the magnetic field strength? 1=3.0 Tesla, 0= 2.0 Tesla

7. Whether the descriptions of the imaging technique were clear so that it could be reproduced? 1= yes, 0= no

8. Whether statistical results were corrected for multiple comparison? 1= corrected, 0= uncorrected or not applicable

9. Whether the conclusions were consistent with the results obtained, and the limitations were discussed? 1= yes, 0= no

^a^ This study has no control group.

^b^ This study used multivariate SVM analysis for classification.

^c^ Although not available in the article, the medication status can be obtained from the cohort studies using the same patient and control groups.

**Abbreviations:** SVM, support vector machine; NA, not applicable
