# Supplementary material for: The neuro-pathophysiology of temporomandibular disorders-related pain: a systematic review of structural and functional MRI studies
Source: J Headache Pain. 2020 Jun 19;21(1):78. doi: 10.1186/s10194-020-01131-4 (PMC7304152; doi:10.1186/s10194-020-01131-4)
Supplement: Supplementary file 2 — Additional file 2: Table S2. Regions with significant group differences between patients with TMD and controls reported in functional MRI studies included in the present systematic review. Table S3. Regions with significant group differences between patients with TMD and controls reported in structural MRI studies included in the present systematic review. [file 10194_2020_1131_MOESM2_ESM.docx]

**Additional file 2**

**Table S2. Regions with significant group differences between patients with TMD and controls reported in functional MRI studies included in the present systematic review.**

| **Studies** | **Modality** | **Analysis method** | **Correction for multiple comparisons** | **Content** | | | | | |
| --- | --- | --- | --- | --- | --- | --- | --- | --- | --- |
| Kucyi et al. [46] | rs-fMRI | Voxel-wise FC | FWE | Seed region (diameter=6 mm) | MNI (mm) | | | | |
|  |  |  |  | Medial prefrontal cortex | -2 | 58 | -6 |  |  |
|  |  |  |  | Regions with significant greater medial prefrontal cortex FC in TMD patients compared with controls | | | | | |
|  |  |  |  | Region | MNI (mm) | | | *Z* score | Cluster size (voxels) |
|  |  |  |  | Lingual gyrus | 6 | -66 | 6 | 3.74 | NA |
|  |  |  |  | Occipital pole | -2 | -88 | -2 | 3.17 | NA |
|  |  |  |  | Right posterior cingulate cortex/precuneus (ventral) | 22 | -62 | 12 | 3.15 | NA |
|  |  |  |  | Precuneus (dorsal) | 0 | -78 | 42 | 3.11 | NA |
|  |  |  |  | Right retrosplenial cortex | 8 | -50 | -8 | 3.07 | NA |
|  |  |  |  | Regions showing a positive correlation between medial prefrontal cortex FC and pain rumination in TMD patients | | | | | |
|  |  |  |  | Region | MNI (mm) | | | *Z* score | Cluster size (voxels) |
|  |  |  |  | Posterior cingulate cortex | 2 | -36 | 10 | 3.23 | NA |
|  |  |  |  | Right medial thalamus | 4 | -16 | 8 | 3.39 | NA |
|  |  |  |  | Left posterior cingulate cortex/precuneus | -6 | -52 | 18 | 3.54 | NA |
|  |  |  |  | Right medial thalamus | 10 | -24 | 8 | 3.49 | NA |
|  |  |  |  | Midbrain | 2 | -30 | -2 | 3.6 | NA |
|  |  |  |  | Left anterior thalamus | -2 | -6 | 0 | 3.7 | NA |
|  |  |  |  | Right posterior cingulate cortex/precuneus | 4 | -50 | 18 | 3.04 | NA |
|  |  |  |  | Right retrosplenial cortex | 6 | -52 | 8 | 3.13 | NA |
|  |  |  |  | Periventricular gray/periaqueductal gray | 2 | -30 | -6 | 2.55 | NA |
| He et al. [47] | rs-fMRI (before and after 3-month splint treatment) | fALFF | AlphaSim | Regions with significant changes in fALFF between patients and controls and between baseline and post-treatment in TMD patients | | | | | |
|  |  |  |  | Region | Talairach (mm) | | | *T* score | Cluster size (voxels) |
|  |  |  |  | *Baseline: Patients < Controls, which were normalized after treatment* | | | | | |
|  |  |  |  | Left precentral gyrus (BA4) | -38 | -15 | 56 | -3.74 | 20 |
|  |  |  |  | Left supplementary motor area (BA6) | -9 | 6 | 66 | -4.47 | 17 |
|  |  |  |  | Left supplementary motor area (BA6) | -3 | 14 | 52 | -4.29 | 16 |
|  |  |  |  | Left middle frontal gyrus (BA10) | -45 | 22 | 32 | -3.87 | 10 |
|  |  |  |  | Right orbitofrontal cortex (BA47) | 30 | 17 | -19 | -5.27 | 15 |
|  |  |  |  | *Post-treatment patients > Baselines patients* | | | | | |
|  |  |  |  | Left precentral gyrus (BA6) | -24 | -9 | 53 | 5.99 | 9 |
|  |  |  |  | Left posterior insula (BA13) | -36 | -19 | 20 | 6.46 | 16 |
|  |  |  |  | *Post-treatment Patients < Controls* | | | | | |
|  |  |  |  | Right superior parietal lobule (BA7) | 18 | -51 | 66 | -5.18 | 27 |
|  |  |  |  | Left postcentral gyrus (BA5) | -20 | -43 | 63 | -4.76 | 17 |
| He et al. [48] | rs-fMRI | Voxel-wise FC | AlphaSim | Seed region (diameter=3.5 mm) | MNI (mm) | | | | |
|  |  |  |  | Dorsal caudate | ±13 | 15 | 9 |  |  |
|  |  |  |  | Ventral caudate/nucleus accumbens | ±9 | 9 | -8 |  |  |
|  |  |  |  | Dorsal caudal putamen | ±28 | 1 | 3 |  |  |
|  |  |  |  | Ventral rostral putamen | ±20 | 12 | -3 |  |  |
|  |  |  |  | Reduced FC in TMD patients compared to controls | | | | | |
|  |  |  |  | *Seed region* and connected region | MNI (mm) | | | *T* score | Cluster size (voxels) |
|  |  |  |  | *Right dorsal caudate* | | | | | |
|  |  |  |  | Right dorsal putamen | 24 | 12 | 3 | 4.24 | 23 |
|  |  |  |  | *Left dorsal caudate* | | | | | |
|  |  |  |  | Left dorsal putamen | -24 | 3 | 12 | 4.9 | 20 |
|  |  |  |  | *Right ventral caudate/nucleus accumbens* | | | | | |
|  |  |  |  | Left anterior insular | -39 | 15 | -9 | 4.73 | 32 |
|  |  |  |  | Left anterior cingulate cortex | -6 | 33 | 21 | 4.31 | 30 |
|  |  |  |  | Right anterior cingulate cortex | 6 | 39 | 9 | 3.92 | 39 |
|  |  |  |  | *Left ventral caudate/nucleus accumbens* | | | | | |
|  |  |  |  | Left putamen | -21 | 9 | 0 | 4.61 | 50 |
|  |  |  |  | Left anterior cingulate cortex | -6 | 33 | 21 | 4.45 | 37 |
|  |  |  |  | *Right dorsal caudal putamen* | | | | | |
|  |  |  |  | Right precentral gyrus | 24 | -18 | 63 | 4.43 | 13 |
|  |  |  |  | Left supramarginal gyrus | -54 | -33 | 15 | 4.1 | 15 |
|  |  |  |  | *Left dorsal caudal putamen* | | | | | |
|  |  |  |  | Left supramarginal gyrus | -54 | -33 | 18 | 4.65 | 10 |
|  |  |  |  | Left precentral gyrus | -48 | 0 | 54 | 4.56 | 10 |
|  |  |  |  | *Right ventral rostral putamen* | | | | | |
|  |  |  |  | Left dorsal caudate | -12 | 12 | 9 | 4.58 | 25 |
|  |  |  |  | Right thalamus | 9 | -15 | 9 | 4.21 | 16 |
|  |  |  |  | Left thalamus | -6 | -12 | 6 | 4.11 | 23 |
|  |  |  |  | *Left ventral rostral putamen* | | | | | |
|  |  |  |  | Left middle temporal gyrus | -57 | -33 | 0 | 4.52 | 26 |
|  |  |  |  | Left dorsal caudate | -12 | 12 | 9 | 3.99 | 17 |
| Zhang et al. [49] | rs-fMRI | ReHo | AlphaSim | Decreased ReHo associated with open-mouth pain in right anterior insular cortex in TMD patients than controls | | | | | |
|  |  |  |  | Region | MNI (mm) | | | *T* score | Cluster size (voxels) |
|  |  |  |  | Right anterior insular cortex | 42 | 9 | 9 | NA | ≥10 |
|  |  | Voxel-wise FC | AlphaSim | Decreased FC associated with open-mouth pain in TMD patients compared with controls using the right anterior insular cortex as a seed | | | | | |
|  |  |  |  | Region | MNI (mm) | | | *T* score | Cluster size (voxels) |
|  |  |  |  | Left mid-cingulate cortex | -15 | -12 | 39 | NA | ≥14 |
|  |  |  |  | Right precuneus | 3 | -69 | 21 | NA | ≥14 |
| Nebel et al. [52] | ts-fMRI (innocuous and low-frequent vibration of index finger) | Brain activation | Cluster-corrected | Regions activated by skin flutter with group differences between TMD patients and controls | | | | | |
|  |  |  |  | Region | MNI (mm) | | | Z score | Cluster size (voxels) |
|  |  |  |  | *Patients < Controls* | | | | | |
|  |  |  |  | Contralateral insula | -44 | -2 | 12 | 5.22 | 145 |
|  |  |  |  | Contralateral S2 parietal operculum subregion 4 | -48 | -4 | 8 | 4.75 |  |
|  |  |  |  | Contralateral S2 parietal operculum subregion 1 | -60 | -22 | 24 | 4.41 | 222 |
|  |  |  |  | Contralateral S1 | -60 | -26 | 46 | 3.82 | 54 |
|  |  |  |  | Contralateral S1 area 1 | -52 | -30 | 56 | 3.63 |  |
|  |  |  |  | *Patients > Controls* | | | | | |
|  |  |  |  | Contralateral thalamus | -8 | -28 | -2 | 4.91 | 501 |
|  |  |  |  | Ipsilateral thalamus | 12 | -24 | 8 | 4.37 |  |
|  |  |  |  | Contralateral S1 area 1 | -54 | -22 | 52 | 4.17 | 102 |
|  |  |  |  | Contralateral S1 area 3b | -46 | -18 | 52 | 3.91 |  |
|  |  |  |  | Contralateral planum temporale | -54 | -30 | 14 | 4.09 | 289 |
|  |  |  |  | Contralateral S2 parietal operculum subregion 1 | -50 | -22 | 14 | 3.96 |  |
|  |  |  |  | Contralateral S2 parietal operculum subregion 1 | -44 | -34 | 20 | 3.93 |  |
|  |  |  |  | Contralateral primary auditory cortex | -44 | -24 | 8 | 3.44 |  |
|  |  |  |  | Ipsilateral primary auditory cortex | 44 | -22 | 6 | 4.57 | 189 |
|  |  |  |  | Ipsilateral S2 parietal operculum subregion 1 | 48 | -24 | 18 | 4.31 |  |
|  |  |  |  | Contralateral insula | -48 | -10 | -8 | 3.86 | 46 |
|  |  |  |  | Contralateral anterior cingulate | -6 | 2 | 40 | 5.1 | 731 |
|  |  |  |  | Ipsilateral anterior cingulate | 4 | 8 | 40 | 4.9 |  |
|  |  |  |  | Contralateral amygdala | -24 | -10 | -12 | 3.94 | 22 |
| Ichesco et al. [51] | rs-fMRI and ts-fMRI (pressure pain to left anterior temporalis) | Voxel-wise FC | AlphaSim | Seed region (diameter=6mm) | MNI (mm) | | | | |
|  |  |  |  | Left anterior insular cortex | -32 | 16 | 6 |  |  |
|  |  |  |  | Left posterior insular cortex | -39 | -15 | 1 |  |  |
|  |  |  |  | Right anterior insular cortex | 32 | 16 | 6 |  |  |
|  |  |  |  | Right posterior insular cortex | 39 | -15 | 8 |  |  |
|  |  |  |  | Increased resting state FC in TMD patients compared to controls | | | | | |
|  |  |  |  | *Seed region* and connectivity region | MNI (mm) | | | *Z* score | Cluster size (voxels) |
|  |  |  |  | *Left anterior insular cortex* | | | | | |
|  |  |  |  | Anterior cingulate cortex (BA24/32) | 2 | 38 | 2 | 4.47 | 101 |
|  |  |  |  | *Left posterior insular cortex* | | | | | |
|  |  |  |  | Left parahippocampal gyrus (BA34) | -14 | -4 | -26 | 5.07 | 176 |
|  |  |  |  | *Right anterior insular cortex* | | | | | |
|  |  |  |  | Right thalamus | 8 | -6 | 6 | 4.35 | 98 |
|  |  |  |  | Greater insular connectivity in TMD patients compared with controls during elicited pain (high pain vs. off) | | | | | |
|  |  |  |  | *Seed region* and connectivity region | MNI (mm) | | | *Z* score | Cluster size (voxels) |
|  |  |  |  | *Left anterior insular cortex* | | | | | |
|  |  |  |  | Anterior cingulate cortex (BA32) | 4 | 42 | 16 | 3.92 | 590 |
|  |  |  |  | Right superior frontal gyrus (BA10/9) | 24 | 52 | 28 | 4.85 | 427 |
|  |  |  |  | Left medial frontal gyrus (BA9/10) | -6 | 56 | 38 | 4.7 | 176 |
|  |  |  |  | *Right anterior insular cortex* | | | | | |
|  |  |  |  | Right anterior cingulate cortex (BA32) | 18 | 38 | 12 | 3.51 | 24 |
| Wessman et al. [54] | ts-fMRI (Stroop task) | Brain activation | AlphaSim | Regions with significant different activations between TMD patients and controls for the neutral Stroop, number counting Stroop, and emotional counting Stroop tasks | | | | | |
|  |  |  |  | Region | Talairach (mm) | | | *T* score | Cluster size (voxels) |
|  |  |  |  | **Neutral Stroop** | | | | | |
|  |  |  |  | *Greater negative activation in patients than controls* | | | | | |
|  |  |  |  | Middle frontal gyrus (BA10, 32) | 8 | 55 | -6 | -6.67 | NA |
|  |  |  |  | Right superior frontal gyrus (BA8) | 17 | 24 | 58 | -5.23 | NA |
|  |  |  |  | Left superior frontal gyrus (BA8) | -26 | 27 | 51 | -6.5 | NA |
|  |  |  |  | Right inferior frontal gyrus (BA10, 47) | 40 | 46 | -1 | -4.43 | NA |
|  |  |  |  | Left inferior frontal gyrus (BA10,47) | -46 | 39 | -1 | 5.92 | NA |
|  |  |  |  | Right middle temporal gyrus (BA21) | 56 | -33 | -3 | -5.86 | NA |
|  |  |  |  | **Number counting Stroop** | | | | | |
|  |  |  |  | *Greater positive activation in patients than controls* | | | | | |
|  |  |  |  | Right middle frontal gyrus (BA9/46) | 47 | 34 | 25 | 4.82 | NA |
|  |  |  |  | Right middle frontal gyrus (BA6, 4) | 49 | 0 | 38 | 6.36 | NA |
|  |  |  |  | Right superior parietal gyrus (BA7) | 17 | -72 | 47 | 8.53 | NA |
|  |  |  |  | Left superior parietal gyrus (BA7) | -21 | -67 | 54 | 8.03 | NA |
|  |  |  |  | Right inferior parietal gyrus (BA40) | 47 | -47 | 43 | 6.56 | NA |
|  |  |  |  | *Less negative activation in patients than controls* | | | | | |
|  |  |  |  | Left pregenual anterior cingulate cortex (BA24, 32) | -2 | 32 | 5 | 6.54 | NA |
|  |  |  |  | Left Amygdala | -17 | -8 | -13 | 4.68 | NA |
|  |  |  |  | *Positive activation in patients while negative activation in controls* | | | | | |
|  |  |  |  | Left subgenual anterior cingulate cortex (BA25) | -5 | 17 | -6 | 4.88 | NA |
|  |  |  |  | Right middle frontal gyrus (BA10) | 40 | 40 | 8 | 5.67 | NA |
|  |  |  |  | Left middle temporal gyrus (BA21) | -50 | -59 | 2 | 5.43 | NA |
|  |  |  |  | Right anterior and middle insula | 37 | -3 | 0 | 5.33 | NA |
|  |  |  |  | *Negative activation in patients while positive activation in controls* | | | | | |
|  |  |  |  | Left middle frontal gyrus (BA6, 4) | -58 | 4 | 11 | 7.05 | NA |
|  |  |  |  | **Emotional counting Stroop** | | | | | |
|  |  |  |  | *Greater positive activation in patients than controls* | | | | | |
|  |  |  |  | Right superior frontal gyrus (BA6) | 5 | -2 | 65 | 6.98 | NA |
|  |  |  |  | Right anterior insula | 32 | 17 | 7 | 5.44 | NA |
|  |  |  |  | *Less negative activation in patients than controls* | | | | | |
|  |  |  |  | Left subgenual anterior cingulate cortex (BA32, 10) | -8 | 33 | -6 | 4.59 | NA |
|  |  |  |  | Right posterior insula | 36 | -7 | -1 | 4.84 | NA |
|  |  |  |  | *Positive activation in patients while negative activation in controls* | | | | | |
|  |  |  |  | Right middle frontal gyrus (BA9, 10) | 37 | 49 | 25 | 6.86 | NA |
|  |  |  |  | Right anterior mid cingulate cortex (BA24) | 3 | 2 | 44 | 4.86 | NA |
|  |  |  |  | Right inferior parietal gyrus (BA40, 7) | 46 | -44 | 44 | 5.98 | NA |
|  |  |  |  | Left inferior parietal gyrus (BA40, 7) | -42 | -48 | 35 | 5.84 | NA |
|  |  |  |  | Left posterior cingulate cortex (BA23, 30, 31) | -4 | -35 | 30 | 7.27 | NA |
|  |  |  |  | Rostral splenial cortex (BA29) | -4 | -45 | 8 | 5.28 | NA |
|  |  |  |  | Caudate nucleus | 4 | 7 | -2 | 4.45 | NA |
|  |  | Brain activation | AlphaSim | Regions with significant different activations between TMD patients and controls evoked by cognitive interference and emotional interference | | | | | |
|  |  |  |  | Region | Talairach (mm) | | | *T* score | Cluster size (voxels) |
|  |  |  |  | **Number Stroop interference (number Stroop minus neutral Stroop)** | | | | | |
|  |  |  |  | *Greater positive activation in patients than controls* | | | | | |
|  |  |  |  | Left inferior parietal gyrus (BA40) | -48 | -33 | 50 | 4.81 | NA |
|  |  |  |  | Left inferior temporal gyrus (BA37) | -50 | -55 | -3 | 4.8 | NA |
|  |  |  |  | *Positive activation in patients while negative activation in controls* | | | | | |
|  |  |  |  | Right superior frontal gyrus (BA10) | 3 | 50 | -10 | 6.76 | NA |
|  |  |  |  | Right pregenual anterior cingulate cortex (BA24/32) | 0 | 36 | 6 | 5.92 | NA |
|  |  |  |  | left precentral gyrus (BA4, 6) | -55 | 3 | 38 | 4.7 | NA |
|  |  |  |  | Left postcentral gyrus (BA3) | -58 | -11 | 38 | 6.18 | NA |
|  |  |  |  | Right amygdala | 28 | -2 | -22 | 5.3 | NA |
|  |  |  |  | Right subthalamic nucleus | 15 | -14 | -6 | 4.34 | NA |
|  |  |  |  | **Emotional Stroop interference (emotional Stroop minus neutral Stroop)** | | | | | |
|  |  |  |  | *Positive activation in patients while negative activations in control* | | | | | |
|  |  |  |  | Left middle frontal gyrus (BA6, 8, 9) | -46 | 3 | 30 | 4.29 | NA |
|  |  |  |  | Left pregenual anterior cingulate cortex and superior frontal gyrus (BA32, 10) | -6 | 61 | 0 | 5.85 | NA |
|  |  |  |  | Left inferior parietal gyrus (BA39) | -41 | -69 | 29 | 6.06 | NA |
|  |  |  |  | Left posterior cingulate cortex (BA23, 31) | -2 | -37 | 32 | 5.26 | NA |
|  |  |  |  | Rostral splenial cortex (BA29) | -4 | -46 | 10 | 5.08 | NA |
|  |  |  |  | Left Middle temporal gyrus (BA21, 37) | -56 | -38 | -9 | 5.78 | NA |
|  |  |  |  | Parahippocampal gyrus | -25 | -19 | -7 | 5.33 | NA |
|  |  | ROI-wise FC | NA | ROI-wise FC analysis: Behavioral performance and connectivity within cognitive and emotional networks | | | | | |
|  |  |  |  | Seed region (diameter=5mm) | Talairach (mm) | | | | |
|  |  |  |  | Right anterior mid-cingulate cortex (BA32) | 7 | 23 | 30 |  |  |
|  |  |  |  | Right superior frontal gyrus (BA9) | 43 | 9 | 33 |  |  |
|  |  |  |  | Pregenual anterior cingulate cortex (BA24/32) | 3 | 40 | 4 |  |  |
|  |  |  |  | Right amygdala | 24 | -4 | -16 |  |  |
|  |  |  |  | During the cognitive interference task, controls showed significant correlations between both 2 pairs of brain areas: aMCC-DLPFC and amygdala-pgACC, while patients did not. | | | | | |
|  |  |  |  | During the emotional interference task, patients showed significant coupling for the amygdala-pgACC pair and for the aMCC-dlPFC pair, while controls did not | | | | | |
| Zhao et al. [53] | ts-fMRI | Brain activation | NA | No group comparison of activated brain regions was performed. | | | | | |
| Gustin et al. [50] | ts-fMRI, DTI, and ASL | Brain activation, FA, and CBF | FWE | TMD showed no S1 functional reorganization, reduced CBF in contralateral S1, or decreased FA in contralateral S1 | | | | | |
| Lickteig et al. [60] | ts-fMRI (occlusal tapping movements, before and after 2-week splint therapy) | Brain activation | FWE | ROI analysis: areas involved in the processing of pain (insula, primary and secondary somatosensory cortex, and cingulate cortex) and areas processing sensorimotor control of occlusal movements (primary motor cortex, superior parietal lobe, and cerebellar hemispheres) | | | | | |
|  |  |  |  | Region | MNI (mm) | | | *T* score | Cluster size (voxels) |
|  |  |  |  | *Reduced activation in the “occlusion” task after therapy compared with pretreatment level* | | | | | |
|  |  |  |  | Right cerebellar hemisphere (posterior lobe) | 27 | -57 | -18 | 7.8 | NA |
|  |  |  |  | Right cerebellar hemisphere (Larsell’s HVI, posterior lobe) | 27 | -60 | -18 | 5.53 | NA |
|  |  |  |  | Right anterior insula | 39 | 21 | 3 | 4.94 | NA |
|  |  |  |  | *Regions with decreased activation in the “occlusion” task after therapy showing positive correlation with pain reduction* | | | | | |
|  |  |  |  | Right anterior insula | 33 | 18 | -21 | 5.42 | NA |
|  |  |  |  | Left posterior insula | -36 | -18 | 18 | 4.98 | NA |
|  |  |  |  | Left cerebellum (crus I, post. lobe) | -18 | -84 | -30 | 5.78 | NA |
|  |  |  |  | Left cerebellum (crus II) | -15 | -84 | -30 | 4.61 | NA |
|  |  |  |  | *Regions with decreased activation showing positive correlation with increased symmetry of condylar movements* | | | | | |
|  |  |  |  | Left cerebellar hemisphere (Larsell’s HVI) | -12 | -78 | -21 | 6.53 | NA |
|  |  |  |  | Right precentral gyrus | 51 | 3 | 30 | 5.87 | NA |
| He et al. [57] | ts-fMRI (teeth clenching before and after 3-month splint therapy) | Brain activation | AlphaSim | Brain regions showing significant different activations during clenching task between patients at baseline and control | | | | | |
|  |  |  |  | Region | MNI (mm) | | | *T* score | Cluster size (voxels) |
|  |  |  |  | *Decreased positive activation in the patient group before treatment than controls during teeth clenching, which were normalized after treatment* | | | | | |
|  |  |  |  | Left precentral gyrus | -39 | -6 | 39 | 4.49 | 130 |
|  |  |  |  | Left inferior temporal gyrus | -54 | 0 | -36 | 4.35 | 27 |
|  |  |  |  | Right inferior temporal gyrus | 54 | -3 | -36 | 4.28 | 58 |
|  |  |  |  | Left cerebellum | -51 | -66 | -33 | 4.73 | 40 |
|  |  |  |  | *Increased negative activation in the patient group before treatment than controls during teeth clenching, which were normalized after treatment* | | | | | |
|  |  |  |  | Right medial prefrontal cortex | 15 | 60 | 9 | 4.25 | 39 |
|  |  |  |  | *Increased positive activation in the patient group after treatment than controls during teeth clenching* | | | | | |
|  |  |  |  | Right cerebellum | 30 | -63 | -36 | 4.5 | 39 |
| Harper et al. [56] | ts-fMRI (subjectively equated noxious temporalis pressures and thumb pressures) | Brain activation | NA | Brain regions from significant weight vector maps contributing to the performance of the support vector machine, which can determinate the location of experimental pain (face pain vs thumb pain) in patients (75%), but not in controls (55%) | | | | | |
|  |  |  |  | Region | MNI (mm) | | | *T* score | Cluster size (voxels) |
|  |  |  |  | *Face > thumb* | | | | | |
|  |  |  |  | Right superior frontal gyrus | 26 | 48 | 16 | NA | 369 |
|  |  |  |  | *Thumb > face* | | | | | |
|  |  |  |  | Left orbitofrontal cortex | -20 | 56 | -8 | NA | 212 |
|  |  |  |  | Left anterior cingulate cortex | -2 | 26 | 24 | NA | 136 |
|  |  |  |  | Left operculum | -54 | 14 | 14 | NA | 92 |
| Roy et al. [55] | ts-fMRI (grip-force task and pain-eliciting stimulus on forearm) | Brain activation | FWE | Regions identified by the machine learner as those that best separated the patients from the controls during the motor task | | | | | |
|  |  |  |  | Region | MNI (mm) | | | *T* score | Cluster size (voxels) |
|  |  |  |  | *Jaw pain group > Controls* | | | | | |
|  |  |  |  | Ipsilateral thalamus (pulvinar) | 9.7 | -23 | 3.4 | NA | 30 |
|  |  |  |  | Contralateral thalamus (pulvinar) | -6.2 | -32 | -0.6 | NA | 14 |
|  |  |  |  | Ipsilateral inferior frontal gyrus (dorsal anterior insula) | 36.8 | 14.9 | 10 | NA | 30 |
|  |  |  |  | Contralateral inferior frontal gyrus (dorsal anterior insula) | -5.1 | 14.7 | -4.7 | NA | 79 |
|  |  |  |  | Contralateral insula (ventral anterior insula) | -42 | -3.6 | -8.2 | NA | 15 |
|  |  |  |  | Ipsilateral middle frontal gyrus (dorsal anterior insula) | 20.3 | 66.3 | 12.7 | NA | 22 |
|  |  |  |  | Ipsilateral middle frontal gyrus (dorsal anterior insula) | 47.4 | 40.5 | 24.8 | NA | 18 |
|  |  |  |  | Ipsilateral precentral gyrus (ventral premotor cortex) | 55.8 | 10.1 | 11.3 | NA | 17 |
|  |  |  |  | Ipsilateral inferior frontal gyrus (ventral premotor cortex) | 56 | 16 | 29.5 | NA | 12 |
|  |  |  |  | Contralateral precentral gyrus (ventral premotor cortex) | -58 | 11.4 | 22.5 | NA | 10 |
|  |  |  |  | Contralateral superior frontal gyrus (supplementary motor area) | -2.3 | -9.1 | 54.2 | NA | 222 |
|  |  |  |  | Contralateral anterior prefrontal cortex (dorsal medial prefrontal cortex) | -1.6 | 55.7 | 16.5 | NA | 149 |
|  |  |  |  | Contralateral superior frontal gyrus (dorsolateral prefrontal cortex) | -24 | 42.8 | 42.1 | NA | 43 |
|  |  |  |  | Contralateral superior frontal gyrus (dorsolateral prefrontal cortex) | -16 | 53.4 | 36.9 | NA | 19 |
|  |  |  |  | Contralateral precuneus | -3 | -56 | 17.6 | NA | 13 |
|  |  |  |  | Contralateral lateral occipital cortex | -9 | -93 | 22.2 | NA | 12 |
|  |  |  |  | Ipsilateral middle temporal gyrus | 51.1 | -53 | 3.1 | NA | 17 |
|  |  |  |  | *Jaw pain group < Controls* | | | | | |
|  |  |  |  | Contralateral orbitofrontal gyrus | -44 | 40.8 | -12 | NA | 12 |
|  |  |  |  | Contralateral orbitofrontal gyrus | -41 | 54.3 | -6.9 | NA | 10 |
|  |  |  |  | Ipsilateral inferior temporal gyrus | 56.2 | -60 | -13 | NA | 38 |
|  |  |  |  | Contralateral inferior parietal lobule | -41 | -36 | 42.7 | NA | 13 |
|  |  |  |  | Ipsilateral lateral occipital cortex | 23.6 | -89 | -4.8 | NA | 145 |
|  |  |  |  | Ipsilateral cerebellum (Crus I) | 24.3 | -82 | -27 | NA | 16 |
|  |  |  |  | Contralateral cerebellum (VI) | -30 | -64 | -25 | NA | 81 |
|  |  |  |  | Contralateral cerebellum (VII) | -15 | -76 | -51 | NA | 15 |
|  |  |  |  | Regions identified by the machine learner as those that best separated the groups during the pain task | | | | | |
|  |  |  |  | Region | MNI (mm) | | | *T* score | Cluster size (voxels) |
|  |  |  |  | *Jaw pain group < Controls* | | | | | |
|  |  |  |  | Ipsilateral precentral gyrus (ventral premotor cortex) | 55.4 | 12.1 | 26.4 | NA | 71 |
|  |  |  |  | Ipsilateral dorsolateral prefrontal cortex/orbitofrontal gyrus | 40.2 | 48.1 | -8.2 | NA | 45 |
|  |  |  |  | Ipsilateral middle frontal gyrus (dorsolateral prefrontal cortex) | 36.3 | 39 | 27.4 | NA | 37 |
|  |  |  |  | Ipsilateral cerebellum (Crus I) | 33 | -83 | -23 | NA | 30 |
|  |  |  |  | Ipsilateral middle frontal gyrus | 39.1 | 55 | 12 | NA | 20 |
|  |  |  |  | Ipsilateral inferior parietal lobule | 39.6 | -44 | 53.1 | NA | 14 |
|  |  |  |  | Contralateral medioventral occipital cortex | -2.8 | -83 | -17 | NA | 32 |
|  |  |  |  | Contralateral inferior frontal gyrus (insula) | -46 | 14.6 | 2.6 | NA | 24 |
|  |  |  |  | Contralateral posterior cingulate cortex | -2.5 | -30 | 29.3 | NA | 13 |
|  |  |  |  | *Jaw pain group > Controls* | | | | | |
|  |  |  |  | Contralateral medial frontal gyrus/orbitofrontal gyrus | -3.4 | 42.4 | -15 | NA | 15 |
|  |  |  |  | Contralateral parahippocampal gyrus | -30 | -31 | -17 | NA | 14 |
| Youssef et al. [23] | ASL | CBF and brain stem blood flow | FDR | Increased cerebral blood flow in TMD patients compared with controls | | | | | |
|  |  |  |  | Region | MNI (mm) | | | *T* score | Cluster size (voxels) |
|  |  |  |  | Right cerebellar cortex | 10 | -48 | -12 | 4.12 | 12 |
|  |  |  |  | Right premotor cortex | 42 | -6 | 60 | 3.98 | 29 |
|  |  |  |  | Right anterior cingulate cortex | 6 | 40 | 22 | 3.7 | 17 |
|  |  |  |  | Left dorsolateral prefrontal cortex | -32 | 52 | 20 | 4.47 | 34 |
|  |  |  |  | Left dorsal precuneus | -12 | -50 | 66 | 5.14 | 176 |
|  |  |  |  | Left ventral precuneus | -16 | -76 | 34 | 4.68 | 221 |
|  |  |  |  | Left supplementary motor area | -16 | 10 | 66 | 4.3 | 26 |
|  |  |  |  | Left premotor cortex | -32 | -14 | 54 | 4.24 | 13 |
|  |  |  |  | Left globus pallidus | -20 | -18 | 10 | 4.23 | 43 |
|  |  |  |  | Increased brain stem blood flow TMD patients compared with controls | | | | | |
|  |  |  |  | Region | MNI (mm) | | | *T* score | Cluster size (voxels) |
|  |  |  |  | Right caudalis division of spinal trigeminal nucleus | 8 | -44 | -60 | 4.09 | 37 |
|  |  |  |  | Right principal sensory trigeminal nucleus | 18 | -40 | -42 | 3.94 | 40 |
|  |  |  |  | Right ventral trigeminothalamic tract | 12 | -24 | -22 | 4.82 | 136 |
| Gerstner et al. [58] | MRS | Metabolite levels | NA | Measuring the metabolite levels (including glutamate, glutamine, N-acetylaspartate, and choline) in the left and right posterior insula without specifying the coordinates and size | | | | | |
| Harfeldt et al. [59] | MRS | Metabolite levels | Uncorrected | Measuring the metabolite levels (including N-Acetyl-aspartate, total creatine, choline, myo-inositol, glutamate, and the combination of Glu and glutamine) in the left and right posterior insula without specifying the coordinates and size | | | | | |

Abbreviations: TMD, temporomandibular disorders; FWE, family-wise error; FDR, false discovery rate; FC, functional connectivity; NA, not applicable; rs-fMRI, resting-state functional magnetic resonance imaging; ts-fMRI, task-state functional magnetic resonance imaging; MRS, magnetic resonance spectroscopy; ASL, arterial spin labeling; DTI, diffusion tensor imaging; MNI, Montreal Neurological Institute coordinates; fALFF, fractional amplitude of low-frequency fluctuation; ReHo, regional homogeneity; BA, Brodmann’s Area; S1, primary somatosensory cortex; S2, secondary somatosensory cortex; CBF, cerebral blood flow; dlPFC, dorsolateral prefrontal cortex; aMCC, anterior mid-cingulate cortex; pgACC: pregenual anterior cingulate cortex

**Table S3. Regions with significant group differences between patients with TMD and controls reported in structural MRI studies included in the present systematic review.**

| **Studies** | **Modality** | **Analysis method** | **Correction for multiple comparisons** | **Content** | | | | | |
| --- | --- | --- | --- | --- | --- | --- | --- | --- | --- |
| Younger et al. [31] | 3D T1 | Whole brain VBM | FDR | Regions with significant GMV differences between TMD patients and controls | | | | | |
|  |  |  |  | Region | MNI (mm) | | | *T* score | Cluster size (voxels) |
|  |  |  |  | *Patients > Controls* | | | | | |
|  |  |  |  | Right inferior frontal gyrus | 44 | 38 | 4 | 5.54 | 487 |
|  |  |  |  | Right anterior insula | 43 | 14 | -2 | 4.72 | 347 |
|  |  |  |  | Right posterior putamen | 28 | -12 | 10 | 4.53 | 253 |
|  |  |  |  | Left ventral posterior thalamus | -20 | -16 | 3 | 4.91 | 379 |
|  |  |  |  | Right ventral lateral thalamus | 13 | -12 | 2 | 4.24 | 367 |
|  |  |  |  | Right globus pallidus | 19 | -8 | 2 | 5.36 | 166 |
|  |  |  |  | Left trigeminal sensory/motor nucleus | -12 | -35 | -34 | 6.14 | 40 |
|  |  |  |  | Right trigeminal sensory/motor nucleus | 14 | -35 | -35 | 5.91 | 36 |
|  |  |  |  | Right medial lemniscus | 9 | -30 | -34 | 5.91 | 182 |
|  |  |  |  | Left middle cerebellar peduncle | -9 | -17 | -28 | 6.57 | 63 |
|  |  |  |  | Right middle cerebellar peduncle | 7 | -17 | -25 | 6.29 | 76 |
|  |  |  |  | *Patients < Control* | | | | | |
|  |  |  |  | Right primary somatosensory cortex | 38 | -27 | 59 | 4.77 | 162 |
|  |  |  |  | Associations between regional GMV with myofascial TMD disease severity | | | | | |
|  |  |  |  | Region | MNI (mm) | | | *T*score | Cluster size (voxels) |
|  |  |  |  | *Regions with decreased GMV, negatively correlated with pain intensity* | | | | | |
|  |  |  |  | Right dorsal posterior cingulate cortex | 10 | -68 | 15 | 8 | NA |
|  |  |  |  | Right rostral anterior cingulate cortex | 1 | 34 | 21 | 5.02 | NA |
|  |  |  |  | Right superior frontal gyrus | 1 | 27 | 48 | 6.91 | NA |
|  |  |  |  | Left superior temporal gyrus | -56 | -44 | 7 | 6.9 | NA |
|  |  |  |  | *Regions with decreased GMV, positively correlated with pressure algometry* | | | | | |
|  |  |  |  | Left trigeminal nucleus | -14 | -39 | -35 | 5.58 | NA |
|  |  |  |  | Right trigeminal nucleus | 14 | -38 | -35 | 5.93 | NA |
|  |  |  |  | *Regions with increased GMV, positively correlated with illness duration* | | | | | |
|  |  |  |  | Left ventral posterior cingulated | -11 | -47 | 15 | 4.83 | NA |
|  |  |  |  | Right ventral posterior cingulated | 6 | -47 | 15 | 5.25 | NA |
|  |  |  |  | Right hippocampus | 35 | -11 | -22 | 5.01 | NA |
|  |  |  |  | Left midbrain | -10 | -18 | -15 | 4.78 | NA |
|  |  |  |  | Right midbrain | 15 | -20 | -15 | 5.63 | NA |
|  |  |  |  | Right middle cerebellar penduncle | 35 | -11 | -22 | 5.01 | NA |
| Gerstner et al. [32] | 3D T1 | Whole brain VBM | Uncorrected | Region | NA | | | *T* score | Cluster size (voxels) |
|  |  |  |  | Regions with decreased GMV in TMD patients compared with controls | | | | | |
|  |  |  |  | Left inferior frontal gyrus (BA45/46) | -47 | 25 | 19 | 4.86 | 676 |
|  |  |  |  | Left anterior cingulate gyrus/medial frontal gyrus (BA8/9/6/32) | -7 | 21 | 45 | 4.71 | 794 |
|  |  |  |  | Right posterior cingulate gyrus/Precuneus (BA31) | 11 | -42 | 41 | 4.21 | 339 |
|  |  |  |  | Left superior temporal gyrus (BA22) | -49 | 5 | -3 | 4.03 | 322 |
|  |  |  |  | Right parahippocampal gyrus (BA36) | 32 | -18 | -25 | 3.88 | 394 |
|  |  |  |  | Right superior temporal gyrus/middle temporal gyrus (BA21) | 51 | -7 | -12 | 3.75 | 268 |
|  |  |  |  | Right anterior insular cortex (BA47/13) | 29 | 23 | -8 | 3.47 | 320 |
|  |  |  |  | Regions with decreased white matter volume in TMD patients compared with controls | | | | | |
|  |  |  |  | Left medial frontal gyrus/superior frontal gyrus/anterior cingulate gyrus | -16 | 25 | 40 | 4.79 | 4133 |
|  |  |  |  | Left precuneus | -17 | -82 | 36 | 4.56 | 526 |
|  |  |  |  | Right inferior frontal/precentral gyrus | 59 | 3 | 22 | 4.37 | 575 |
|  |  |  |  | Right medial frontal gyrus/superior frontal gyrus/anterior cingulate gyrus | 18 | 25 | 43 | 4.26 | 2007 |
|  |  |  |  | Left middle frontal gyrus | -35 | 26 | 29 | 4.26 | 622 |
|  |  |  |  | Regions with increased white matter volume in TMD patients compared with controls | | | | | |
|  |  |  |  | Right superior temporal gyrus/supramarginal gyrus | 43 | -52 | 20 | 5.07 | 2887 |
|  |  |  |  | Left superior temporal gyrus/middle temporal gyrus | -35 | -56 | 19 | 4.96 | 1090 |
| Gustin et al. [35] | 3D T1 | Whole brain VBM | FDR | TMD patients showed no significant differences in regional GMV and N-acetylaspartate/creatine in thalami compared with controls. | | | | | |
|  | MRS | Metabolite levels | NA |  |  |  |  |  |  |
| Moayedi et al. [39] | 3D T1 | CTA (ROI) | Bonferroni correction | Regions with increased cortical thickness in TMD patients compared with controls | | | | | |
|  |  |  |  | Region | Talairach (mm) | | | *T* score | Cluster size (vertices) |
|  |  |  |  | Right primary somatosensory cortex (BA2) | 50 | -18 | 37 | 5.18 | 1132 |
|  |  |  |  | Left frontal polar cortex (BA10) | -30 | 45 | 6 | 4.87 | 569 |
|  |  |  |  | Left ventrolateral prefrontal cortex (BA9/10) | -38 | 35 | 4 | 3.41 | 531 |
|  |  |  |  | Cortical thickness negatively correlates with TMD pain intensity or unpleasantness | | | | | |
|  |  |  |  | Region | Talairach (mm) | | | T score | Cluster size (vertices) |
|  |  |  |  | *Pain intensity* | | | | | |
|  |  |  |  | Left anterior mid-cingulate cortex (BA32) | -4 | 18 | 19 | -3.94 | 718 |
|  |  |  |  | Left primary motor cortex (BA4) | -57 | -3 | 13 | -4.15 | 674 |
|  |  |  |  | *Unpleasantness* | | | | | |
|  |  |  |  | Left orbitofrontal cortex (BA11/47) | -27 | 16 | -19 | -4.22 | 472 |
|  |  | VBM (ROI) | FDR | TMD duration is positively correlated to GMV in sensory thalamus | | | | | |
|  |  |  |  | Region | Talairach (mm) | | | *T* score | Cluster size (voxels) |
|  |  |  |  | Left thalamus posterior nucleus | -14 | -26 | 12 | 7.84 | 2312 |
|  |  |  |  | Left thalamus ventrolateral nucleus | -14 | -14 | 17 | 5.7 |  |
|  |  |  |  | Left thalamus ventroposterior medial nucleus | -12 | -16 | 9 | 5.12 |  |
|  |  |  |  | Right ventroposterior medial nucleus | 15 | -19 | 14 | 4.21 | 14 |
| Moayedi et al. [41] | 3D T1 | CTA (ROI) | AlphaSim | Age-related group differences in cortical thickness | | | | | |
|  |  |  |  | Region | Talairach (mm) | | | *T* score | Cluster size (vertices) |
|  |  |  |  | *Patients < Controls* | | | | | |
|  |  |  |  | Right anterior mid-cingulate cortex /pregenual anterior cingulate cortex | 14 | 30 | 20 | 4.42 | 116 |
|  |  |  |  | *Patients > Controls* | | | | | |
|  |  |  |  | Right premotor cortex | 8 | 12 | 54 | -5.18 | 109 |
|  |  | VBM (ROI) | FDR | Age-related group differences in subcortical GMV | | | | | |
|  |  |  |  | Region | Talairach (mm) | | | *T* score | Cluster size (voxels) |
|  |  |  |  | *Patients > Controls* | | | | | |
|  |  |  |  | Left dorsal striatum | -18 | 10 | 10 | -4.99 | 1537 |
|  |  |  |  | Right dorsal striatum | 11 | -3 | 18 | -4.65 | 4415 |
|  |  |  |  | Left thalamus | -21 | -23 | 14 | -3.36 | 264 |
| Salomons et al. [43] | 3D T1 | CTA (ROI) | AlphaSim | Region | MNI (mm) | | | *r* value | Cluster size (vertices) |
|  |  |  |  | Regional cortical thickness in TMD patients correlated with helplessness | | | | | |
|  |  |  |  | *Positive correlation* | | | | | |
|  |  |  |  | Supplementary motor area | -9 | -10 | 55 | 0.69 | NA |
|  |  |  |  | *Negative correlation* | | | | | |
|  |  |  |  | Mid-cingulate cortex | -5 | -2 | 31 | -0.80 | NA |
|  |  |  |  | Posterior cingulate cortex | -5 | -23 | 32 | -0.79 | NA |
|  |  | FA (TBSS) | AlphaSim | Regional FA in TMD patients correlated with helplessness | | | | | |
|  |  |  |  | *Positive correlation* | | | | | |
|  |  |  |  | Cingulum | 10 | -33 | 40 | 0.61 | 21 |
|  |  |  |  | Superior corona radiata (BA5) | 15 | -49 | 61 | 0.8 | 25 |
|  |  |  |  | Corticospinal tract | -11 | -31 | 55 | 0.61 | 14 |
|  |  |  |  | Corticospinal tract (BA6) | -10 | -27 | 53 | 0.73 | 15 |
|  |  |  |  | *Positive correlation* | | | | | |
|  |  |  |  | Splenium of corpus callosum | -17 | -33 | 30 | -0.60 | 38 |
|  |  |  |  | Body of corpus callosum | 20 | -31 | 36 | -0.58 | 88 |
|  |  |  |  | Corticospinal tract (midbrain) | 17 | -10 | -4 | -0.63 | 17 |
|  |  |  |  | Corticospinal tract (posterior limb of the internal capsule) | 23 | -5 | 19 | -0.69 | 134 |
|  |  |  |  | Corticospinal tract (BA6) | -10 | -21 | 65 | -0.69 | 36 |
|  |  |  |  | Corticospinal tract (pons) | 9 | -20 | -25 | -0.67 | 30 |
| Wilcox et al. [45] | 3D T1 | VBM (ROI) | NA | TMD patients had no change in volume or DTI values in the trigeminal root entry zone compared with controls. | | | | | |
|  | DTI | FA and MD |  |  |  |  |  |  |  |
| Wilcox et al. [22] | 3D T1 | VBM (ROI) | FDR | Intergroup Comparison for VBM and DTI | | | | | |
|  |  |  |  | Region | MNI (mm) | | | Z score | Cluster size (voxels) |
|  |  |  |  | *Grey matter volume: Patients < Controls* | | | | | |
|  |  |  |  | Ipsilateral spinal trigeminal nucleus | -4 | -46 | -66 | 4.27 | 113 |
|  |  |  |  | Rostral medullary raphe | -1 | -39 | -51 | 3.63 | 34 |
|  |  |  |  | Ipsilateral principle trigeminal nucleus | -13 | -43 | -38 | 3.92 | 115 |
|  | DTI | FA and MD | Small volume correction | *Mean diffusivity: Patients > Controls* | | | | | |
|  |  |  |  | Ipsilateral spinal trigeminal nucleus | -6 | -42 | -64 | 3.36 | 79 |
|  |  |  |  | Ipsilateral trigeminal nerve tract | -21 | -33 | -35 | 2.88 | 13 |
|  |  |  |  | Contralateral trigeminal nerve tract | 18 | -30 | -34 | 3 | 36 |
|  |  |  |  | Periaqueductal grey matter | -1 | -34 | -8 | 3.73 | 98 |
| Moayedi et al. [42] | DTI | ROI (FA, MD, and RD) | NA | TMD patients had lower FA, higher MD and RD in left and right trigeminal nerve root (manually drew ROIs, 3.75 mm × 3.75 mm × 3 mm) compared with controls. | | | | | |
|  |  | Voxel-wise analysis | NA | Compared with controls, TMD patients had reduced FA in the whole brain and within the white matter skeleton, as well as higher MD, RD, and axial diffusivity within the white matter skeleton. | | | | | |
|  |  | FA (TBSS) | Threshold-free cluster enhancement | White matter regions in TMD patients with significantly lower FA compared to controls identified within the white matter skeleton mask | | | | | |
|  |  |  |  | Region | MNI (mm) | | | *T* score | Cluster size (voxels) |
|  |  |  |  | Thalamus | 17 | -15 | 12 | 4.08 | 305 |
|  |  |  |  | White matter adjacent to primary somatosensory cortex/primary motor cortex | 32 | -22 | 38 | 3.51 | 129 |
|  |  |  |  | Anterior limb of the internal capsule | 14 | 12 | -2 | 3.06 | 3 |
|  |  |  |  | External/extreme capsules | 33 | 6 | 3 | 2.9 | 4 |
|  |  |  |  | External/extreme capsules | 32 | 9 | 1 | 2.82 | 3 |
|  |  |  |  | Internal capsule | 11 | 1 | 3 | 2.6 | 581 |
|  |  |  |  | Anterior limb of the internal capsule and external/extreme capsules | 26 | 24 | 11 | 2.27 | 55 |

Abbreviations: TMD, temporomandibular disorders; FDR, false discovery rate; ROI, regions of interest; NA, not applicable; 3D T1, three-dimensional T1- weighted anatomical image; BA, Brodmann’s Area; MNI, Montreal Neurological Institute coordinates; VBM, voxel-based morphometry; GMV, grey matter volume; CTA, cortical thickness analysis; FA, fractional anisotropy; MD, mean diffusivity; RD, radial diffusivity; TBSS, tract-based spatial statistics
